# Non-governmental organization facilitation of a community-based nutrition and health program: Effect on program exposure and associated infant feeding practices in rural India

**DOI:** 10.1371/journal.pone.0183316

**Published:** 2017-09-14

**Authors:** Veena Singh, Saifuddin Ahmed, Michele L. Dreyfuss, Usha Kiran, Deepika N. Chaudhery, Vinod K. Srivastava, Ramesh C. Ahuja, Abdullah H. Baqui, Gary L. Darmstadt, Mathuram Santosham, Keith P. West

**Affiliations:** 1 Center for Human Nutrition, Department of International Health, Johns Hopkins Bloomberg School of Public Health, Baltimore, Maryland, United States of America; 2 Department of Population and Family Health, Johns Hopkins Bloomberg School of Public Health, Baltimore, Maryland, United States of America; 3 CARE-India, New Delhi, India; 4 King George Medical University, Lucknow, India; 5 Health Systems Division, Department of International Health, Johns Hopkins Bloomberg School of Public Health, Baltimore, Maryland, United States of America; TNO, NETHERLANDS

## Abstract

**Background:**

Integrated nutrition and health programs seek to reduce undernutrition by educating child caregivers about infant feeding and care. Data on the quality of program implementation and consequent effects on infant feeding practices are limited. This study evaluated the effectiveness of enhancing a nutrition and health program on breastfeeding and complementary-feeding practices in rural India.

**Methods:**

Utilizing a quasi-experimental design, one of the implementing districts of a Cooperative for Assistance and Relief Everywhere (CARE) nutrition and health program was randomly selected for enhanced services and compared with a district receiving the Government of India’s standard nutrition and health package alone. A cohort of 942 mother-child dyads was longitudinally followed from birth to 18 months. In both districts, the evaluation focused on responses to services delivered by community-based nutrition and health care providers [anganwadi workers (AWWs) and auxiliary nurse midwives (ANMs)].

**Findings:**

The CARE enhanced program district showed an improvement in program coverage indicators (e.g., contacts, advice) through outreach visits by both AWWs (28.8–59.8% vs. 0.7–12.4%; all p<0.05) and ANMs (8.6–46.2% vs. 6.1–44.2%; <0.05 for ages ≥6 months). A significantly higher percentage of child caregivers reported being contacted by the AWWs in the CARE program district (20.5–45.6% vs. 0.3–21.6%; p<0.05 for all ages except at 6months). No differences in ANM household contacts were reported. Overall, coverage remained low in both areas. Less than a quarter of women received any infant feeding advice in the intervention district. Earlier and exclusive breastfeeding improved with increasing number or quality of visits by either level of health care provider (OR: 2.04–3.08, p = <0.001), after adjusting for potentially confounding factors. Socio-demographic indicators were the major determinants of exclusive breastfeeding up to 6 month and age-appropriate complementary-feeding practices thereafter in the program-enhanced but not comparison district.

**Interpretation:**

An enhanced nutrition and health intervention package improved program exposure and associated breastfeeding but not complementary-feeding practices, compared to standard government package.

**Trial registration:**

ClinicalTrials.gov NCT00198835

## Introduction

Poor feeding during the first two years of life is common among undernourished children in South Asia [1,2]. Educating parents and other child-care givers about nutritious, affordable and hygienic infant feeding practices is expected to improve children’s nutritional status [3]. However, little is objectively known about what strategies are the most effective, when implemented, in infant and child feeding and counseling programs as infants grow or how variations in exposure to various components of such programs actually influence infant feeding practices and subsequent health outcomes.

Efficacy trials in Mexico and Bangladesh demonstrated that interventions including home-based nutrition education and peer-counseling, when adequately delivered, can improve rates of breastfeeding [4] and complementary feeding [5] of young children. A few studies have also shown that educating child caregivers on feeding practices can improve program coverage [6–9] when properly implemented properly at a small scale. However, there is little evidence of effectiveness of these interventions in changing home feeding behavior and improving child nutritional status when they are scaled-up to large programs [10–13]. With a rising global emphasis on achieving improvements in nutritional outcomes at scale, there is an urgent need to evaluate the impact as well as the processes of implementation and spread of large-scale nutrition programs.

In India, the Integrated Child Development Services (ICDS) and Reproductive and Child Health (RCH) programs are the largest state programs in the world, reaching an estimated 8 million pregnant and lactating women and 39 million children under six years of age [14]. Interventions are designed to improve nutrition and health of mothers and young children. These interventions are provided through their respective community-based health care providers, the anganwadi workers (AWWs) who are at least a high school graduate, and auxiliary nurse midwives (ANMs) who are trained nurse practitioner. Both cadres of staff are responsible for educating child-caregivers about breastfeeding and complementary feeding practices, vaccination and delivery of supplementary nutrition. In addition, they are responsible for assuring vaccination compliance and delivering supplementary foods intended to support early preschool child growth on a regular basis. While these program’s and services are longstanding, there remains a relative paucity of data clearly linking the level and continuity of services provided and their corresponding effects on the nutritional and health status of children.

The present comparative cohort study in two districts in India provided a unique opportunity to evaluate effects of ICDS and RCH program exposure, assessed with respect to coverage, intensity and quality of service provision by AWWs and ANMs on infant breastfeeding and complementary feeding practices.

## Evaluation design and study setting

### Evaluation design

In both the Intervention and comparison districts, three rural blocks were purposively selected. Block population size and number of Anganwadi Centers (AWCs) were also considered in selecting comparable blocks in the 2 districts. Only the blocks where Cooperative for Assistance and Relief Everywhere (CARE) India was actively implementing INHP-II replication sites were considered in the intervention district. In the comparison district, the comparability of a block's population size and number of functioning AWC—a central place for service delivery in each village for community-based health workers—was considered in addition to the other criteria mentioned above. There were 6–7 sectors per block in the intervention district and 5–6 sectors per block in the comparison district. Four sectors were randomly selected from each of the 3 blocks in each district. Altogether, there were 135 AWCs in the intervention and 192 in the comparison district. Finally, 5–7 AWCs per sector from both districts were selected to get the sample size of 81–84 AWCs each for study inclusion. Villages were selected through multi-stage random sampling, in a CARE enhanced intervention district and a non-CARE district with only the Government of India’s (GOI’s) standard of care (i.e. ICDS and RCH programs), respectively. These two study areas were at almost equal distances from the state capital and were comparable in socio-demographic characteristics. Investigators at The Johns Hopkins Bloomberg School of Public Health (JHBSPH), Baltimore, Maryland, USA, in collaboration with King George Medical University (KGMU), Lucknow, India, designed and implemented this study. Role of CARE-India was limited to program implementation, updating investigators regarding various programmatic components, and providing lists of intervention villages.

### Study setting

The study was carried out in two rural districts in the state of Uttar Pradesh, from May 2004 to July 2006. Despite decades of programming efforts, most of the maternal and childcare indicators in the state of Uttar Pradesh remain lower than the national average. For example, only a quarter of women receive at least three antenatal care check-ups during pregnancy (26.6% in Uttar Pradesh vs. 52% nationally), fewer women have institutional deliveries (20.6% vs. 38.7%) and only 14.9% (vs. 41.2%) have a postnatal check-up. A lower proportion of women under the ICDS umbrella in UP actually receive any services under the program (62.6% vs. 72.4% nationally). Where fewer than 21% of women in the nation reported any specific maternal care services during pregnancy or lactation from the Anganwadi center (AWC), this coverage was under 10% in the state of Uttar Pradesh. While in the populous state of Uttar Pradesh where 18.7% of the nation’s 136.1 million children under the age of six reside [15], less than a quarter of children utilized any childcare services (22.3% in UP vs. 32.9% nationally) such as supplementary food, complete immunization, or weighing during health check-ups^2^.

### Role of funding source

The study was funded by the USAID, India Mission, through Global Research Activity Award # HRN-A-00-96-90006-00 to the Johns Hopkins Bloomberg School of Public Health, MD, USA. VS was supported during analysis and reporting through Gates Grant GH 614 and the George G. Graham Professorship Endowment at Johns Hopkins University. The funders had no role in study design, data collection and analysis, decision to publish, or preparation of the manuscript. The corresponding author had full access to all the data in the study and final responsibility for the decision to submit the paper for publication.

## Service provider responsibilities

### Comparison program/ Standard nutrition and health programs (ICDS and RCH)

AWWs are primarily responsible for providing supplementary nutrition to pregnant and lactating women and children under six, and educating preschool children through the ICDS program. In coordination with ANMs, AWWs also provide nutrition and health-care education, health check-ups and referral services. The ANMs alone have been the focus of RCH programs and are responsible for providing antenatal care, delivery care, and immunization services. At the time of the study, an AWW was expected to cover a population of approximately 2–3,000, however, most covered a larger population. Likewise, an ANM was responsible for covering a population of 5–7,000 through a sub-center, but in most cases exceeded this level. The AWWs are expected to deliver services during monthly growth monitoring contacts with caregivers of under-two children whereas ANMs provide services during visits for childhood immunization.

### Intervention program/ Enhanced Integrated Nutrition and Health Program (INHP II)

CARE-India, under its second phase of INHP umbrella adopted a demonstration and replication approach for scaling up the successful practices through government systems. Ten percent of the sites, where CARE worked with local non-government organization (NGOs) and community-based organization (CBOs), were developed as demonstration sites to innovate and develop practices that could help solve operational problems faced by the ICDS and RCH programs. These learning laboratories for government counterparts helped identify and demonstrate effectiveness of existing best practices in solving operational problems and to generate convincing evidence to take the lessons to scale. The key program inputs for translating and replicating doable actions at system and community level included;

#### Capacity building

The primary tool to take intervention to scale included, technical content, process skills and motivation during formal training; and, cross-visits, joint program reviews, supportive supervision and on-going capacity building through review meetings.

#### Behavior change communication (BCC)

Formative research conducted in the initial phase across states. It helped identify barriers and was useful in the development of culturally appropriate communication materials for different contexts. The BCC approach complemented the capacity building efforts. It was expected to bring about changes in behaviors at the household level through use of multiple channels of communication, including mainly, the most important of which being—interpersonal communication.

#### System strengthening

The approach focused on building capacities within the ICDS and RCH programs to sustain changes brought about by INHP II. Specifically, this meant helping the ICDS and RCH programs improve the food and health supply chain for more effective delivery of services, strengthening the information systems for better monitoring and influencing the training content. INHP-II placed significant level of effort and resources into strengthening the food supply chain management to improve the supplementary feeding component of ICDS.

**Fig 1:**
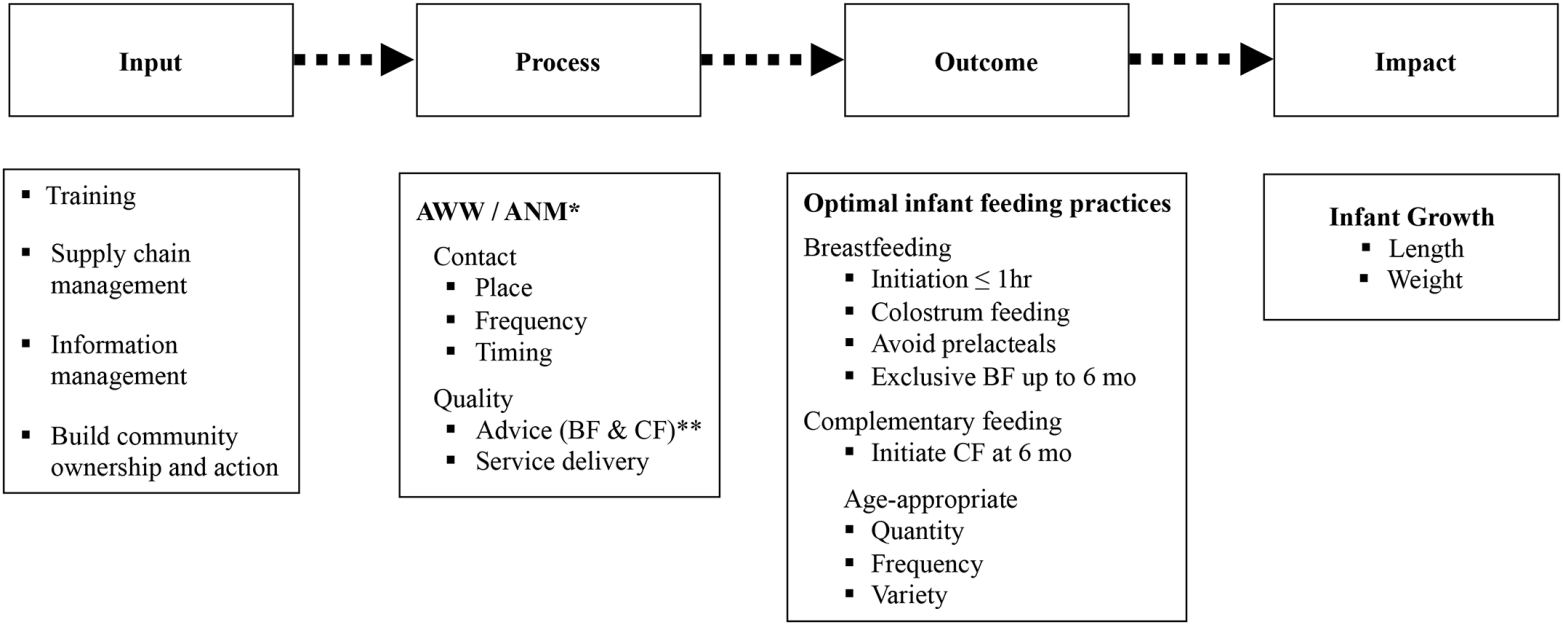
Program impact pathway (PIP) of CARE-India’s enhanced package. *AWW: Anganwadi Worker; ANM: Auxiliary Nurse Midwife ** BF: Breastfeeding; CF: Complementary Feeding.

Once, ‘best practices’ were established as solutions to specific operational problems at the demonstration sites, INHP II was implemented at scale in replication sites. The implementation included; capacity building through technical inputs and training; behavior change communication through increased equitable and improved coverage along with more frequent contacts; strengthening key systems (eg. supply chain management, and information management); and building community ownership and action. The involvement of the GOIs the ICDS and RCH programs in this approach was integral. The AWWs and ANMs, and their supervisors at sector and block levels, had assigned roles in the execution of each best practice. The service providers were trained and encouraged to contact pregnant women and child caregivers at pre-specified time, place and frequency. At each visit, pre-defined nutrition and health care components of the package were emphasized (Fig 1).

Under the enhanced package protocol, AWWs were expected to contact pregnant women early in the pregnancy and advise them regarding optimal breastfeeding practices. For the first 28 days after birth, the AWWs were expected to visit the homes regularly and advise mothers regarding breastfeeding. During months 1–6, the AWW was expected to make at least 12 contacts (twice monthly visits) to each home, advising mothers regarding exclusive breastfeeding and initiation of complementary feeding at 6 months of age. During months 7–8, AWWs were expected to make almost daily contact at each home to establish complementary feeding. From months 9–24, the AWWs were expected to make twice monthly home visits to advise the mothers to continue on-demand breastfeeding and age appropriate complementary feeding.

The ANMs were expected to make at least 3 contacts with each mother during pregnancy at the health center, to be present during deliveries, and to make at least one postnatal home visit to promote vaccination. In the first 6 months, ANMs were expected to make monthly contact with lactating mothers; from months 7–8, they were expected to visit sick children at home; and in months 9–24 they should have performed home visits of sick children and provided vitamin A supplementation and pediatric iron-folic acid (IFA) supplementation from month 12 onwards (Table 1).

**Table 1:** Protocol for place, timing and frequency of community-based nutrition and health care provider contacts within CARE-India’s Integrated Nutrition and Health Package (INHP II)^1^.

Life cycle period	Number of contacts and services delivered	
	Anganwadi Worker (AWW)	Auxiliary Nurse Midwife (ANM)
Pregnancy	3 Home/ other visits to advise on breastfeeding	3 contacts–monthly
Birth—28 days	5 Visits, same breastfeeding goals	2 contacts—Delivery and 1 home visit for vaccination advice
1–6 months	12 Visits, same breastfeeding goals plus initiation of complementary feeding at 6 months of age	6 contacts at health center
7–8 months	Daily visits to establish complementary feeding	Monthly clinic contact and home visits to sick children
9–11 months	4 Visits, same breastfeeding and complementary feeding goals	Same monthly clinic and home visits, plus vitamin A supplements
12–24 months	24 Visits, same breastfeeding and complementary feeding goals	Same monthly clinic and home visits plus vitamin A and iron-folic acid supplements

^1^ Applies to CARE’s INHP II (Integrated Nutrition and Health Program–“Intervention district”).

Since, the study aimed to evaluate the effectiveness of INHP II interventions at-scale, the evaluation was carried out in the replication sites where the program was primarily delivered through the existing systems and structures of the GOIs, the ICDS and RCH functionaries. The INHP II acted as an external catalyst and facilitator for making these programs sharper, more focused and effective by providing the necessary technical, managerial and resource support.

## Sample size & data collection

Sample size was calculated to detect the proportional difference in infant nutritional status at 6, 12 and 18 months of age between the intervention and the comparison districts. The sample size prevalence estimates were based on NFHS-3, Uttar Pradesh, India 2005–06 report. This sample size was also adequate to detect differences in infant dietary intake outcomes between the intervention and comparison districts.

District program officers and chief medical officers were contacted before starting the study to fully brief them about the study and obtain approvals. Village heads were contacted for oral permission and written informed consent was obtained from each study participant.

Third trimester pregnant women identified during house-to-house surveys were enrolled in the study. Women were considered eligible if they were at least 24 weeks of gestation on the day of house-listing and intended to remain in the program area (same village) for the duration of the study or were permanently residing (>1 year) with their parents. Gestational age was assessed through recall of the date of the last menstrual period (LMP) and inquiries about the number of completed months of pregnancy.

**Fig 2:**
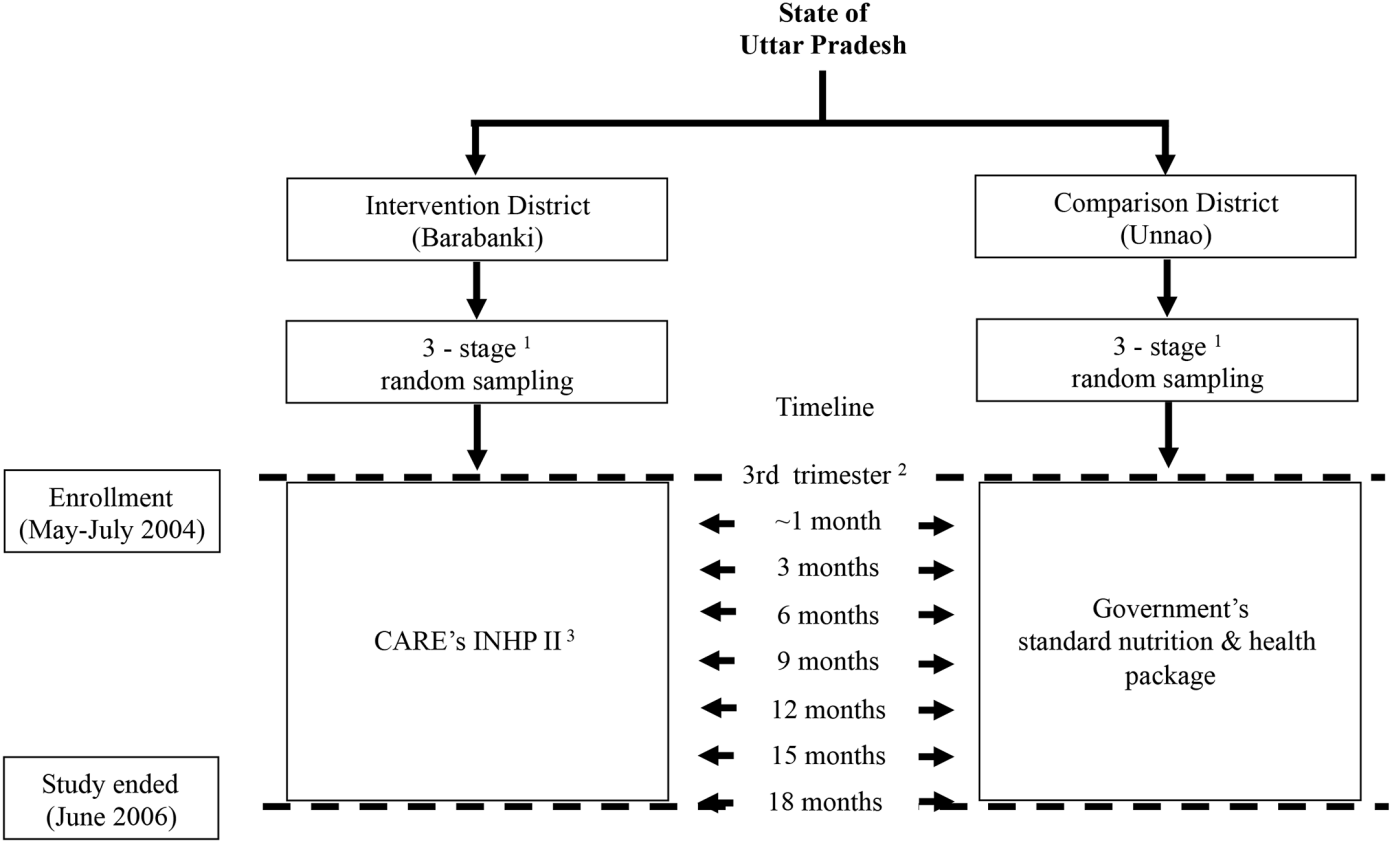
Flow chart of the design and timeline of evaluation visits by the research team in the intervention and comparison districts. ^1^ A multi-stage random sampling strategy consisted of selection of three blocks each, followed by 4 sector per block, and 5–7 anganwadi worker coverage areas per sector in the intervention and comparison districts ^2^ Women were enrolled during the 3^rd^ trimester of pregnancy from May to July 2004 ^3^ CARE’s INHP II program included the Government of India’s nutrition and health package enhanced by intensive service provider training and supportive supervision ^4^ Follow-up ended at 18 months of age or by June 2006, whichever time came first.

Evaluation visits were scheduled to capture the expected service provider contacts, nutrition and health services utilized, and infant feeding advices received. The first follow-up visit was scheduled 7 days after delivery with a visit every 3 months thereafter until the infant reached the age of 18 months or the study ended. During the enrollment visit (6–9 months gestation), socio-demographic and birth preparedness information was also collected. At the 7-days postpartum visit, data were collected on the delivery experience and breastfeeding initiation. During post-partum visits, mothers were asked about immunizations and infant feeding practices. The extent of service provider contact and health service utilization was assessed during antenatal visits and at every follow-up visit (Fig 2).

The institutional review board at the Johns Hopkins Bloomberg School of Public Health (JHBSPH), Baltimore, MD and the ethical committee at King Georges Medical University (KGMU), Lucknow, UP approved the study. This was an ‘exempt’ trial that was not a "clinical trials" that require clinicaltrials.gov registration. The initial IRB approval from the JHBSPH was obtained on August 1, 2003. In 2005, the International Committee of Medical Journal Editors (ICMJE) began to require trial registration as a condition of publication. Therefore, to get this registered, IRB approval from the KGMU was obtained on December 28, 2004 and the trial was registered in 2005. The authors confirm that all ongoing and related trials for this drug/intervention are registered.

## Data review and analysis

The quality of data was checked and validated at the project office in Lucknow. In the cases of discrepancies, data were crosschecked with the paper documentation for immediate resolution if possible. After assessing the data for quality, frequency distributions of the sample characteristics were examined with descriptive statistics. Indicators were compared using Student’s t-test and Pearson’s chi-square for metric (continuous) and non-metric (categorical) variables, respectively. All the women enrolled in their third trimester of pregnancy with a live birth and interviewed within a month after delivery and every three months thereafter were included in this analysis.

At various time intervals, program exposure and infant feeding outcome indicator variables were generated. Three primary program exposure indicators at various ages; coverage—service provider contacts, intensity—number of contacts with the service providers, and quality–advice received, were generated. Indicators for initiation of breastfeeding at ~ one month, infant feeding at 6 months of age and age-appropriate complementary feeding practices (9–18 month) were generated. Infant feeding practices indicators comprised of: exclusive breastfeeding up to 6 months of age and initiation of complementary feeding after 6 months, and assessment of age-appropriate complementary feeding practices such as–the quantity, frequency and variety of complementary food at the respective ages. A woman was considered to have received an antenatal check-up if she had her blood pressure taken and/or an abdominal examination performed.

Trend tests were performed to determine whether breastfeeding initiation practices systematically increased or decreased with the quality and/or intensity of program coverage. The trend test is an extension of the Wilcoxon rank-sum test and tests the alternative hypothesis that outcomes systematically increase or decrease over the ordered levels of the program exposure indicator variable. Logistic regression with population-averaged option was used to assess the outcomes of breastfeeding initiation and infant feeding practices at 6 months of age. Population-averaged logit models [xtlogit y x1 x2 x3……..xn, i(awc) pa] were used to account for study design and to analyze repeated program exposure and complementary feeding outcomes assessed at 9, 12, 15 and 18 months of age.

Xt series of commands provide tools for Population-averaged (PA) model:

xtlogit y x1 x2 x3……..xn, i(awc) pa

This is equivalent to xtgee models with family(binomial), link(logit) with corr(exchangeable). The standard errors are somewhat larger than those obtained without the vce(robust) option because the output includes the additional panel-level variance component. This is parameterized as the log of the variance ln(σ^2^_ʋ_) (labeled lnsig2u in the output). The standard deviation σ_ʋ_ is also included in the output and labeled sigma u together with ρ (labeled rho),

ρ(rho) = σ2ʋ /σ2ʋ+ σ2Є

which is the proportion of the total variance contributed by the panel-level variance component. When rho is zero, the panel-level variance component is unimportant, and the panel estimator is no different from the pooled estimator. A likelihood-ratio test of this is included at the bottom of the output. This test formally compares the pooled estimator (logit) with the panel estimator.

## Results

To identify and enroll third trimester pregnant women in our study, 1,330 and 1,217 pregnant women were identified; and, 512 and 468 pregnant women were enrolled in the intervention and the comparison districts respectively. These eligible women were screened and were contacted 7 days after delivery and the mother-child dyads were followed through 18 month of age (consort flowchart: Paper 1: simultaneously published manuscript titled: An integrated nutrition and health program package on IYCN improves breastfeeding but not complementary feeding and nutritional status in rural northern India: a quasi-experimental randomized longitudinal study).

Socio-demographic characteristics of mothers assessed at the time of enrollment was reported previously (Paper 1: submitted together) and showed no statistically significant (p> 0.05) difference between the intervention and the comparison district in most of the assessed indicators except Muslim religion (23.6% vs. 5.6%), schedule caste/ tribe (37.0% vs. 43.1%), >20 years of age (84.4% vs. 79.5%), working outside the home (32.0% vs. 39.3%), receiving an antenatal check-up (42.3% vs. 31.3%) and institutional delivery (18.9% vs. 9.0%).

### Program exposure

#### Coverage /Service provider contacts

A significantly higher percentage of participants in the intervention district reported having contacts with either of the service providers (Table 2). Coverage by the AWWs in the intervention district ranged from about 30%-60% across time periods, whereas fewer than 22% of study participants in the comparison area reported having any contacts with an AWW. When stratified by the place of contact, intervention district participants reported higher contact at all time periods (ranging from 20–45% vs. <1–18%). There were significantly higher contacts with ANMs at the ages of 6–18 months. For the earlier ages (<6 months of age), there were no reported differences in service provider contacts.

**Table 2:** Program coverage and places of contacts with community-based health care providers as reported by study participants for infant feeding advice and delivery of other services at various time periods in rural Uttar Pradesh, India (2004–06).

Places of contact	Timing of evaluation visits													
	3^rd^ trimester		Postpartum											
			~1 mo		3 mo		6 mo		9 mo		12 mo		18 mo	
	Inter. (n = 492)^1^	Comp. (n = 450)^1^	Inter. (n = 420)	Comp. (n = 375)	Inter. (n = 418)	Comp. (n = 387)	Inter. (n = 414)	Comp. (n = 370)	Inter. (n = 391)	Comp. (n = 348)	Inter. (n = 367)	Comp. (n = 298)	Inter. (n = 340)	Comp. (n = 297)
**Anganwadi worker**														
Home visit % (n)	33.9(167)	17.8*(80)	28.8(121)	4.8*(18)	45.6(190)	8.8*(34)	35.6(147)	5.7*(21)	20.5(80)	2.6*(9)	26.7(98)	0.3*(1)	24.4(83)	0.3*(1)
Other place % (n)	-	-	0.5(2)	0.3(1)	29.2(122)	4.9*(19)	24.2(100)	6.5*(24)	14.8(58)	2.3*(8)	14.4(53)	1.0*(3)	14.1(48)	0.3*(1)
Any contact^2^ % (n)	50.8(250)	21.6*(97)	28.8(121)	5.1*(19)	59.8(250)	12.4*(48)	47.5(196)	11.4*(42)	29.2(114)	4.9*(17)	34.9(128)	1.3*(4)	32.7(111)	0.7*(2)
**Auxiliary nurse midwife**														
Home visit % (n)	24.2(119)	29.6(133)	7.4(31)	11.2(42)	28.2(118)	32.3(125)	23.2(96)	21.6(80)	8.7(34)	12.6(44)	9.5(35)	8.4(25)	8.5(29)	3.0*(9)
Other place % (n)	-	-	1.4(6)	1.3(5)	31.1(130)	18.1*(70)	28.3(117)	9.7*(36)	20.7(81)	5.2*(18)	10.4(38)	3.4*(10)	8.5(29)	3.0*(9)
Any contact^2^ % (n)	50.4(248)	55.6(250)	8.6(36)	12.0(45)	46.2(193)	44.2(171)	45.4(188)	30.3*(112)	27.9(109)	17.8*(62)	18.8(69)	11.7*(35)	15.9(54)	6.1*(18)

Inter. = Intervention district, Comp. = Comparison district

* P-value for chi2 test: < 0.05

^1^ 12 and 3 study participants from the intervention and the comparison districts respectively were excluded due to refusal for all the successive visits following child birth

^2^ Any contact includes home visit

#### Intensity/ Dose-delivery

**Fig 3:**
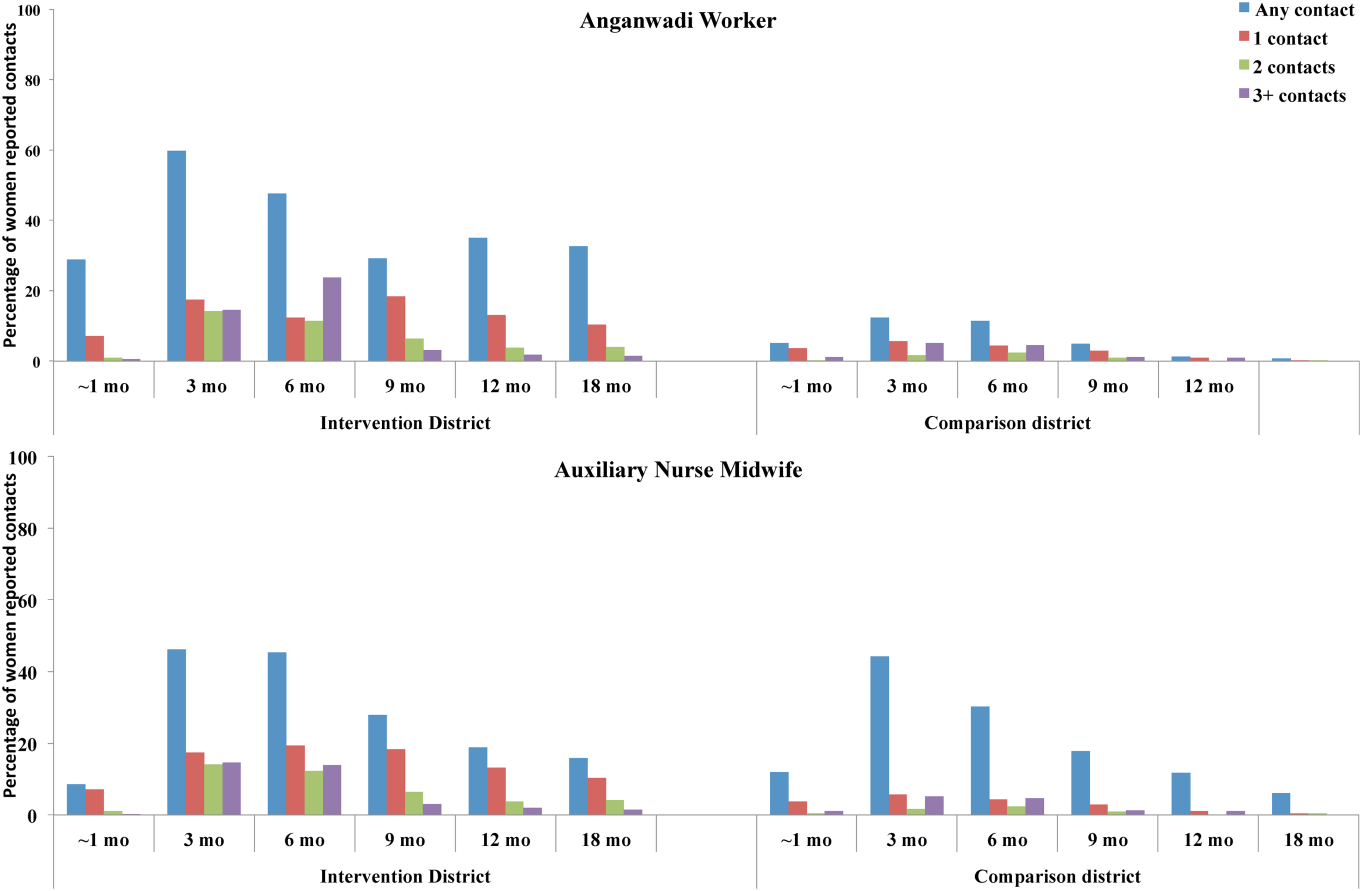
Intensity of any contacts as reported by study participants for infant feeding advice and delivery of other services at different time periods in rural Uttar Pradesh, India (2004).

When stratified by the number of reported contacts, Fig 3 illustrates a significantly increased proportion of 1, 2, or 3+ visits by AWWs in the intervention district at all time periods. Major increases were reported at 3 and 6 months of age. However, significantly increased proportions of 1, 2 and 3+ contacts with ANMs were reported in the intervention district only at visits that occurred from month 6 onwards. At earlier visits, there were no differences in the intensity of contact between the intervention and the comparison districts.

#### Quality of contacts

The quality of contact was defined by the contacts with the service providers and advice received and/or services received during that contact as reported by the mother/child caregiver. Approximately 30%, 60% and 50% of the study participants in the intervention district reported being contacted by an AWW at the 1^st^, 3^rd^ and 6-month evaluation visits; but, only 21%, 23% and 6% of the women, respectively, received any breastfeeding advice in the comparison district (Table 3). In the comparison district, <12% of the participants had any contact with the AWW and <2% reported receiving breastfeeding advice. The quality of contact was significantly higher in the intervention than the comparison area at all three time periods, although less than a quarter of study participants reported being advised regarding breastfeeding practices (Table 4). Less than 8% of participants in either of the study areas reported any breastfeeding advice from the ANM.

**Table 3:** Education about breastfeeding (BF) practices by study participants (by recall) during time intervals prior to evaluation visits in rural Uttar Pradesh, India (2004–06).

Quality of service: Breastfeeding	Timings of postpartum evaluation visits					
	~1 mo		3 mo		6 mo	
	Inter.^1^ (n = 420)	Comp.^1^ (n = 375)	Inter. (n = 418)	Comp. (n = 387)	Inter. (n = 414)	Comp. (n = 370)
**Anganwadi worker**						
No BF advice % (n)	7.9 (33)	2.9 (11)*	36.8 (154)	11.4 (44)*	39.0 (161)	10.8 (40)*
BF advice % (n)	21.4 (90)	2.1 (8)	23.0 (96)	1.0 (4)	8.5 (35)	0.5 (2)
Not contacted % (n)	71.0 (298)	94.9 (356)	40.2 (168)	87.6 (339)	52.5 (217)	88.6 (327)
**Auxiliary nurse midwife**						
No BF advice % (n)	4.8 (20)	10.9 (41)*	38.3 (160)	41.6 (161)*	40.8 (169)	29.2 (108)*
BF advice % (n)	3.8 (16)	1.1 (4)	7.9 (33)	2.6 (10)	4.6 (19)	1.1 (4)
Not contacted % (n)	91.4 (384)	88.0 (330)	53.8 (225)	55.8 (216)	54.6 (226)	69.7 (258)

Int. = Intervention district, Comp. = Comparison district

* P-value for chi2 test: < 0.05

^1^ 12 and 3 study participants from the intervention and the comparison districts respectively were excluded due to refusal for all the successive visits following child birth

**Table 4:** Recall of advice about complementary feeding (CF) practices by study participants during time intervals prior to evaluation visits in rural Uttar Pradesh, India (2004–06).

Quality of service: Complementary feeding	Timings of postpartum evaluation visits							
	6 mo		9 mo		12 mo		18 mo	
	Inter. (n = 414)	Comp. (n = 370)	Inter. (n = 391)	Comp. (n = 348)	Inter. (n = 367)	Comp. (n = 298)	Inter. (n = 340)	Comp. (n = 297)
**Anganwadi worker**								
No CF advice % (n)	34.6 (143)	10.6 (39)*	13.8 (54)	4.9 (17)*	8.7 (32)	1.0 (3)*	6.5 (22)	0.3 (1)*
CF advice % (n)	12.8 (53)	0.8 (3)	15.4 (60)	0	26.2 (96)	0.3 (1)	26.2 (89)	0.3 (1)
Not contacted % (n)	52.5 (217)	88.6 (327)	70.8 (277)	95.1 (331)	65.1 (239)	98.7 (294)	67.4 (229)	99.3 (295)
**Auxiliary nurse midwife**								
No CF advice % (n)	39.4 (163)	29.2 (108)	20.5 (80)	17.0 (59)*	16.1 (59)	11.7 (35)*	12.1 (41)	6.1 (18)*
CF advice % (n)	6.0 (25)	1.1 (4)	7.4 (29)	0.9 (3)	2.7 (10)	0	3.8 (13)	0
Not contacted % (n)	54.6 (226)	69.7 (258)	72.1 (282)	82.2 (286)	81.2 (298)	88.3 (263)	84.1 (286)	93.9 (279)

Int. = Intervention district, Comp. = Comparison district

* p-value for chi2 test: < 0.05

Among mothers in the intervention district who reported being contacted by the AWW from 6 to 18 months (30–50%) after childbirth, only 13% and 15% reported having received complementary feeding advice at 6 and 9 months, and ~26% at 12 and 18 months, respectively (Table 4). In contrast, <1% of the participants received any complementary feeding advice in the comparison areas. The difference in the quality of contact with the AWW, in terms of receiving complementary feeding advice, between the intervention and the comparison districts was significant at all time periods. Specific advice on complementary feeding by the ANM was uniformly low (3%-7% in the intervention and <1% in the comparison district), although differences in the quality of contact were significant at 9, 12 and 18 months of age.

### Services utilization

The proportion of participants who reported receiving vitamin A supplements during the 9 to 18 month visits ranged from 18% to 70% in the intervention district and from 15% to 35% in the comparison district, respectively (Table 5). Receipt of pediatric IFA ranged from 14% to 67% and supplementary food from 41% to 55% in the intervention district, as opposed to the comparison district where <1% of the participants received pediatric iron-folic acid (IFA) supplements and receipt of supplementary food ranged from 4%-22%. Overall, the differences in the proportions of participants who received vitamin A, pediatric IFA and food supplements were significant at all ages.

**Table 5:** Health services received from any health care provider as reported by study participants at various time periods in rural Uttar Pradesh, India (2004–06).

Services received^1^	Approximate timings of postpartum evaluation visits							
	9 mo		12 mo		15 mo		18 mo	
	Inter.	Comp.	Inter.	Comp.	Inter.	Comp.	Inter.	Comp.
	(n = 367)	(n = 298)	(n = 367)	(n = 298)	(n = 297)	(n = 223)	(n = 340)	(n = 297)
Vitamin A supplementation % (n)	17.7 (69)	14.7 (51)	41.1 (151)	25.2 (75)*	66.3 (197)	35.0 (78)*	70.3 (239)	32.0 (95)*
Pediatric iron folic-acid % (n)	-	-	14.2 (52)	0.3 (1)*	59.0 (175)	0.5 (1)*	66.5 (226)	1.4 (4)*
Supplementary nutrition % (n)	-	-	40.6 (149)	4.4 (13)*	51.5 (153)	6.3 (14)*	55.0 (187)	21.9 (65)*

Inter. = Intervention district, Comp. = Comparison district

* p-value for chi2 test: < 0.05

^1^ Includes auxiliary nurse midwife, anganwadi worker but may include any other government health care provider (eg, at primary health center or sub-center etc

The proportion of pregnant women who received four ante-natal check ups, as recommended by the WHO clinical guidelines, remained low (12.7% vs. 9.05%) in the intervention and comparison areas. A significantly higher proportion of women received any antenatal care check-up in the intervention areas (41.66% vs. 31.57%) (Table 6).

**Table 6:** Number of ante-natal care received as reported by study participants in rural Uttar Pradesh, India (2004–06).

Number of ante-natal visits received	Project Districts		p-value^1^
	Intervention (Barabanki) (n = 504)	Comparison (Unnao) (n = 453)	
	% (n)	% (n)	
1	12.70 (64)	12.36 (56)	0.355
2	11.11 (56)	7.28 (33)	
3	5.16 (26)	2.87 (13)	
4+	12.70 (64)	9.05 (41)	
No visit	58.33 (294)	68.42 (310)	

^1^ Statistical testing by chi square for contingency presentations, with r-1 x c-1 degrees of freedom where r = number of comparison groups (r = 2) and c = number of strata for each comparison (c = x)

### Determinants of infant feeding practices

#### Breastfeeding initiation (at ~1 month)

**Fig 4:**
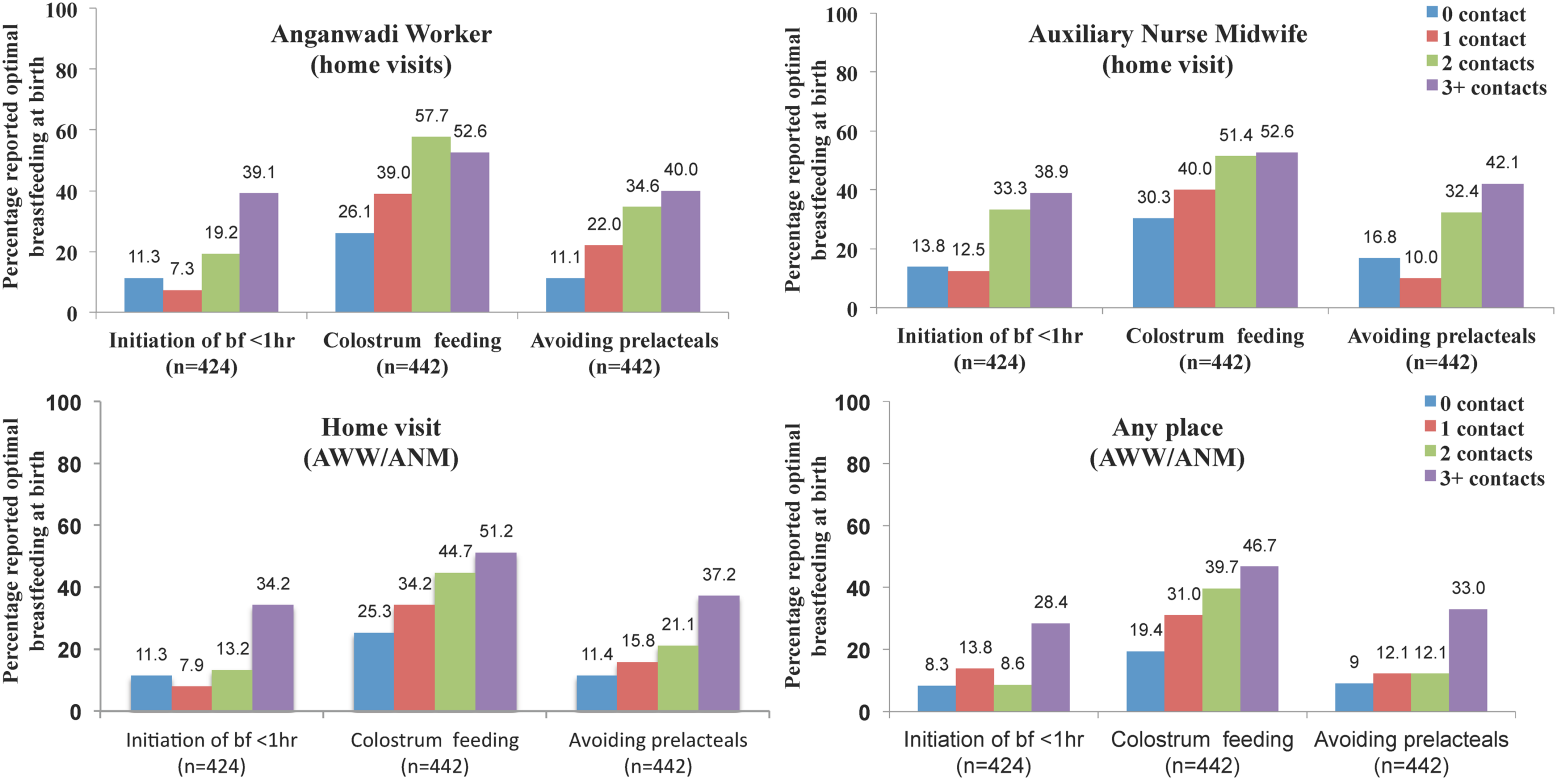
Service provider contacts and breastfeeding practices at the time of birth as obtained at the ~1 month post-partum evaluation visit. ^1^ From the test, there appears to be a trend in breastfeeding practices across the ordered levels of ANM contact from no contact, to contact only and contact along with maternal feeding advice ^2^ Avoiding prelacteal feeding include water, ghutti, jeera, pudina, honey, sugar, jaggary, juice, tea, coffee or any other watery liquids.

**Fig 5:**
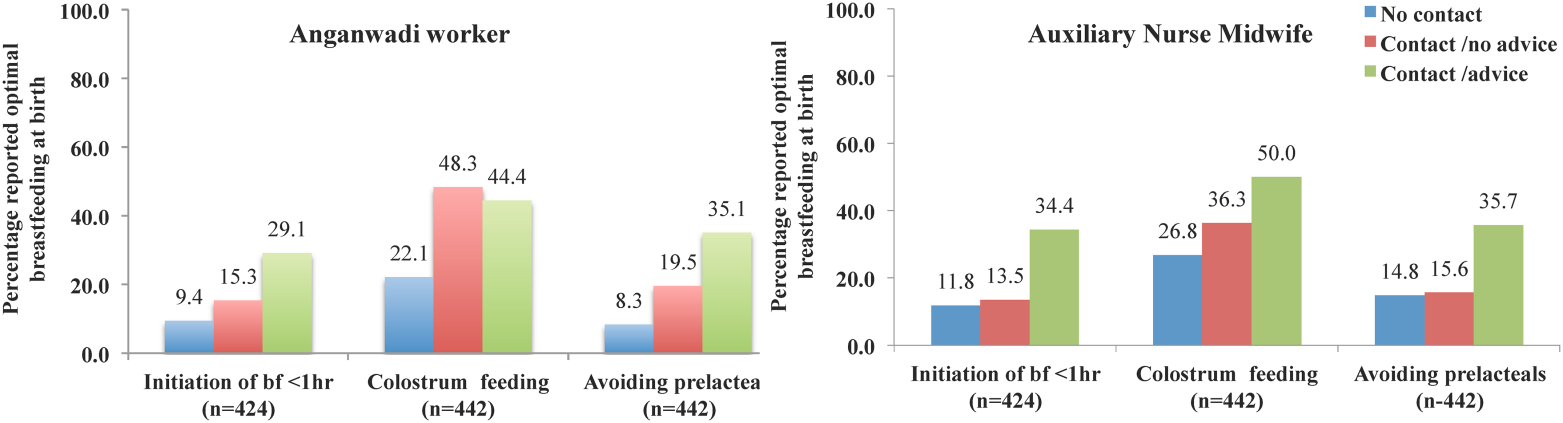
Contact and advice by anganwadi workers and auxiliary nurse midwives during pregnancy and breastfeeding practices at the time of birth as obtained at the ~1 month post-partum evaluation visit. ^1^ From the statistical test for trend, there appears to be a trend in breastfeeding practices (p<0.001) across the ordered levels of ANM contact from no contact, to contact only and contact along with maternal feeding advice ^2^ Avoiding prelacteal feeding include water, ghutti, jeera, pudina, honey, sugar, jaggary, juice, tea, coffee or any other watery liquid.

Trend tests to determine whether breastfeeding initiation practices systematically increased or decreased over the intensity or quality of the visits by either of the service care providers showed significant positive associations between breastfeeding initiation indicators and number of contacts with either of the service providers regardless of place of contact (Fig 4). Initiation of breastfeeding within 1 hour of childbirth, feeding colostrum and avoiding pre-lacteals also showed strong positive associations with the quality of contacts with AWW and ANM (Fig 5).

In bivariate analyses, all program exposure indicator variables for health worker contacts and health services utilization demonstrated significantly higher odds of breastfeeding initiation practices. Among socio-demographic indicators, only religion was highly significantly associated with avoiding pre-lacteals, and marginally with colostrum feeding; paternal education and institutional delivery showed marginally significant association with avoiding pre-lacteals. However, in the multivariate analysis, only home visits by AWWs (breastfeeding initiation (OR): 2.04, 95% CI: 1.20, 3.45; colostrum feeding (OR): 2.69, 95% CI: 1.74, 4.15; avoiding pre-lacteals (OR): 1.73, 95% CI: 1.73, 5.47), receipt of antenatal check-ups (breastfeeding initiation (OR): 1.87, 95% CI: 1.09, 3.22; colostrum feeding (OR): 1.43, 95% CI: 1.01, 2.04; avoiding pre-lacteals (OR): 1.98, 95% CI: 1.17, 3.35) and supplementary food (breastfeeding initiation (OR): 2.47, 95% CI: 1.31, 4.65; avoiding pre-lacteals (OR): 2.71, 95% CI: 1.19, 6.17) remained significant after adjusting for maternal education and caste (Table 7).

**Table 7:** Adjusted odds of optimal breastfeeding initiation practices and infant feeding practices at 6 months of age in rural Uttar Pradesh, India (2004–06).

Characteristics	Breastfeeding initiation practices			Infant feeding practices	
	Initiation of BF < = 1 hr	Colostrum feeding	Avoiding pre-lacteals	Exclusive breastfeeding up to6 mo	Initiation of complementary feeding after 6 mo
**Socio-demographic variables**					
Maternal education	1.45 (0.77, 2.73)	1.03 (0.62, 1.70)	1.16 (0.63, 2.15)	2.15 (1.17, 3.96)*	2.42 (1.49, 3.96)***
Caste					
General/others	1.0	1.0	1.0	1.0	1.0
OBC	1.34 (0.50, 3.61)	0.79 (0.39, 1.60)	0.78 (0.32, 1.95)	0.38 (0.14, 1.04)	0.69 (0.36, 1.03)
SC/ST	2.47 (0.91, 6.71)	1.07 (0.53, 2.17)	1.23 (0.46, 3.24)	0.23 (0.08, 0.66)**	0.54 (0.27, 1.07)
Pre-term births^1^	-	-	-	0.39 (0.21, 0.72)**	-
**Health worker contacts**					
Home visit by anganwadi worker	2.04 (1.20, 3.45)**	2.69 (1.74, 4.15)***	3.08 (1.73, 5.47)***	0.61 (0.36, 1.03)	-
Any contact with auxiliary nurse midwife	-	-	-	-	-
**Health services utilization**					
Antenatal care check-up	1.87 (1.09, 3.22)*	1.43 (1.01, 2.04)*	1.98 (1.17, 3.35)*	-	1.53 (1.01, 2.33)*
Receipt of:					
Supplementary nutrition	2.47 (1.31, 4.65)**	-	2.71 (1.19, 6.17)*	-	-
Tetanus toxoid vaccination	-	-		-	-

p-value for significant at:

*p<0.05.

**p<0.01 and

***p<0.001

^1^ Calculated from last menstrual period (LMP) recall as reported by the mother at the time of enrollment and infant’s date of birth

In the comparison district, maternal education, caste and place of delivery showed significant association with breastfeeding initiation practices in bivariate analysis; however, no variable showed any significant association in the multivariate analysis. For the outcome of avoiding pre-lacteals, the effect sizes were too small to perform multivariate analysis.

#### Infant feeding (at 6 month)

Relative odds of exclusive breastfeeding at 6 months of age in the intervention district was significantly higher for the participants of a higher wealth quintile, educated parents, general caste and stay-at-home mothers. Among the program exposure variables, a home visit by any service provider and an antenatal check-up during pregnancy were significantly associated with exclusive breastfeeding at 6 months of age. In multivariate analysis, the odds of exclusive breastfeeding up to 6 months were 2.15 (95% CI: 1.17, 3.96) for infants of literate mothers, while study participants belonging to other backward caste or schedule caste/tribe or with preterm birth were less likely to exclusively breastfeed their infants in the intervention areas. None of the program exposure variables showed a significant association with exclusive breastfeeding up to 6 months of age. A similar trend was observed for the initiation of complementary feeding at 6 months where only maternal education (OR: 2.42, 95% CI: 1.49, 3.96) remained significant in multivariate analysis (Table 7). Similar analysis in the comparison area demonstrated that none of the socio-demographic or program exposure variables had any significant association with either of the outcomes in the multivariate analysis.

#### Complementary feeding (6–18 month)

The odds of feeding age-appropriate quantities of complementary foods were significantly associated with the father’s education, caste and maternal gravidity (having a previous child) in the intervention district whereas maternal education and any contact with the AWW were significantly associated with reported consumption of age-appropriate frequency of complementary feeding (Table 8). Reported age-appropriate consumption of a variety of complementary foods was significantly positively associated with parental education and any contact with the AWW. No single variable demonstrated any significant association with any of the age-appropriate complementary feeding practices in the comparison district.

**Table 8:** Adjusted odds of age-appropriate optimal complementary feeding practices at 9, 12, 15 and 18 month of age in rural Uttar Pradesh, India (2004–06).

Characteristics	Age appropriate complementary feeding		
	Quantity	Frequency	Variety
**Socio-demographic variables**			
Maternal education	0.72 (0.50, 1.03)	0.73 (0.55, 0.97)*	1.34 (1.00, 1.79)*
Paternal education	0.56 (0.38, 0.82)**	**-**	1.48 (1.11, 1.96)**
Caste			
General/others	1.0	1.0	1.0
OBC	1.67 (1.07, 2.59)*	0.79 (0.52, 1.19)	1.05 (0.71, 1.5)
SC/ST	2.94 (1.75, 4.94)**	1.15 (0.73, 1.81)	-
Infant’s gender			
Male	1.36 (0.98, 1.88)	-	-
Parity	1.67 (1.13, 2.47)*	-	-
**Health worker contacts**			
**Anganwadi worker**			
Home visit	-	-	-
Any contact	0.72 (0.51, 1.01)	0.74 (0.56, 0.98)*	1.32 (1.00, 1.73)*
**Auxiliary nurse midwife**			
Home visit	-	-	-
Any contact	-	-	-

p-value for significant at:

*p<0.05.

**p<0.01 and

***p<0.001

## Discussion

The INHP II program was successful in improving optimal breastfeeding practices from generally very modest to higher levels that are likely to have nutritional benefits. However, as there were few differences in complementary feeding practices and overall nutritional status, it appears that early, more complete breastfeeding achieved under program conditions may not improve these facets of early life nutrition (Paper 1: simultaneously published manuscript titled: An integrated nutrition and health program package on IYCN improves breastfeeding but not complementary feeding and nutritional status in rural northern India: a quasi-experimental randomized longitudinal study). Additionally, many outcomes/other practices may differentially improve healthy growth. Thus, it was imperative to understand the program implementation and determinants of feeding practices that were relevant to the context.

The critical path hypothesized to improve infant feeding and growth was through improving the effectiveness of the existing system; namely, timing and frequency and quality of service provider contacts and service delivery. The INHP II significantly improved the AWW contacts including home visits, and showed moderate improvement in contacts with the ANMs at 6 months and beyond. Similarly, intensity of contact with the AWW at home or any other place remained significantly better in the intervention district than the comparison district. Further, quality of contact with the AWW, in terms of reported breastfeeding advice, though significantly better in the intervention district, remained low. Quality of contact with the ANMs, though significantly better in the intervention district, remained low and did not have had any effect on infant feeding practices. Overall, less than a quarter of study participants, in either of the district, received any complementary feeding advice from either of the service provider.

Besides, AWWs and ANMs, another cadre of community-based volunteers called ‘change agents’ (CAs), were trained. The CAs represented and catered to a cluster of 15–25 households, working with support of the AWWs to improve child health and nutrition practices were trained. However, less than 5% women reported having any contact with the CAs. Therefore, this component of the INHP II program was dropped after mid-term evaluation by CARE-India. Still, the contribution of the CAs in program effectiveness/coverage, at least in the beginning of the study, could not be ruled out.

Frequency and quality of contacts with either of the provider was positively associated with indicators of breastfeeding initiation. This was further observed when multivariate analysis was performed. For the outcomes assessed at 6 months of age, socio-demographic variables, including maternal education and caste, were strong determinants for exclusive breastfeeding. A community-based randomized efficacy trial of breastfeeding promotion in Mexico City showed significant increase in breastfeeding exclusivity and duration with early and repeated contact with peer counselors [4]. Similar trends were observed in rural Haryana, India where repeated counseling using multiple channels improved exclusive breastfeeding at 3 month, complementary feeding and energy intake [6–7]. However, use of local NGO workers in mobilizing the community was acknowledged in this study. Lack of such facilitation, in scaled-up phase may not show similar results.

In a cluster randomized trial in peri-urban areas of Peru, more child caregivers in the intervention area reported receiving nutrition advice and were more likely to feed nutrient dense thick food to their infants at 6 month of age [8]. A community-based weaning intervention in rural Bangladesh, where community-based volunteers were trained to teach families regarding complementary feeding, more infants in the intervention group reported consuming significantly greater percentage of protein and energy from complementary food 5. Though varying in design and scale, these studies show consistent improvement in feeding practices with increased coverage, intensity and quality of contact [5–8].

Given the scale of the present study and low numbers of child-caregivers receiving feeding advice, a socio-demographic factor,—maternal education showed increased odds of timely initiation of complementary feeding at 6 month of age. No difference between the intervention and the comparison districts in initiation of complementary feeding was observed. Similarly, complementary feeding indicator showed that father’s education, caste and having a previous child were the strongest determinants of age-appropriate feeding. Parental education and any contact with the AWW were significantly associated with feeding of age-appropriate frequency and variety of complementary foods. The infant’s gender and religion did not appear to influence any of the infant feeding practices. Maternal age categories, parity and childbirth interval were also not associated with any of the infant feeding outcomes.

The decreased odds of frequency but increased odds of the variety of complementary feeding could be attributed to seasonal availability of local food as program exposure between the intervention and the comparison district at later ages remained relatively low.

Utilization of health care services, as assessed through reception of supplemental vitamin A, consumption of pediatric IFA and supplementary nutrition, showed significant improvement in CARE enhanced areas. Still, only half to two third of the study participants received these services in the intervention areas whereas it was almost non-existent in the comparison areas. The difference in services, primarily provided by the ANMS, namely, vitamin A supplementation and ante-natal care check show significantly lower difference in service delivery than pediatric IFA and supplementary nutrition where AWWs and support staff (‘dai’/sahayika) may provide additional assistance in service delivery.

As of December 2004, there were 5652 ICDS functional projects in the country, 4533 in rural, 759 in tribal and 360 in urban areas [16]. While ICDS had focused on children of 3–6 years at its inception, as more evidence was generated, the focus was re-directed to pregnancy and the first 2–3 years of a child’s life. However, attention was more focused on increasing program coverage and providing supplementary nutrition [17]. With recent evidence highlighting that nutrition education is more crucial than food supplementation/insecurity for combatting undernutrition among preschoolers, the present study fills a crucial gap in the knowledge base for program implementation and outcome measures [18].

The program was unique in its implementation strategy as the ICDS and RCH functionaries of the GOI were responsible for the delivery of services through existing structure and system. Although, CAs were trained and employed in the beginning, the component was removed after mid-term evaluation. Program also evolved with respect to strengthening supervisory system, synergizing efforts at sector level, structuring to hands on training and problem solving, focusing on select interventions, using the monitoring data for feedback, re-thinking geographical focus and re-defining CARE’s personnel’s roles.

Similarly, strength of this study included, large cohort, villages as the unit of selection, randomization in selection of AWCs, use of open ended questionnaire with respect to age-appropriate breastfeeding and complementary feeding advice received at various ages and implementation of identical evaluation tools in both the districts. To best of our understanding, this study was first of its kind and may help improve our understanding of the ‘program exposure’ and its relationship with the various outcome and impact indicators.

Some of the limitation of this study included lack of control with respect to assignment of intervention and comparison district. The INHP II was already implemented in one of the districts; therefore, we selected a nearby district with the comparable baseline indicators. Secondly, given the nature of the intervention, it was not possible to do blinding with respect to data collection. To overcome this limitation, we switched teams collecting data from one district to other at fixed time intervals. Still, bias in probing by the interviewers could not be ruled out.

In year 2006, the Department of Women and Child Development under the Ministry of Human Resource Development was elevated to the status of an independent Ministry at the Union level^19^. This change in the administrative hierarchy is intended to re-direct attention to children’s issues and affect resource allocation. The Ministry of Women and Child Development is expected to consolidate maternal and child health issues for effective implementation.

Many recent intervention evaluation studies have provided evidence in support of making an intervention more effective by adding additional components to the existing package [19–20]. Although it is a step in the right direction, it once again begs the question, what happens when we scale-up an intervention? Do we have the resources, both human and capital, to integrate these new components? What will happen to the existing package? Is there any way to make the existing package more effective while removing unnecessary components and enforcing the proven effective ones in a given context?

In recent years, many experts have pointed out the importance of generating data to evaluate the effectiveness of interventions. For nutritional interventions, this can be done most efficiently by evaluating existing programs [10, 11]. This study is a step in that direction and it adds to the evidence-base on important programmatic components in scaling up those models in the existing intervention to improve infant feeding practices and ultimately towards growth and health of children.

## Supporting information

S1 File Cohort research protocol. (DOC)

S2 File Relative odds of optimal breastfeeding initiation practices in the intervention district in rural Uttar Pradesh, India (2004–06) p-value for significant at: *p<0.05. **p<0.01 and ***p<0.001 (Table A). Odds of initiation of breastfeeding within < = 1 hour of delivery, feeding colostrum and avoiding pre-lacteals in the comparison district in rural Uttar Pradesh, India (2004–06) ANC: Antenatal checkups received; PHC: Primary Health care center; AWW: Anganwadi worker; P-value for chi2 tests: < 0.05 (Table B). Relative odds of optimal infant feeding practices at 6 month of age in rural Uttar Pradesh, India (2004–06) ANC: Antenatal checkups received; AWW: Anganwadi worker; p-value for significant at: *p<0.05. **p<0.01 and ***p<0.001 (Table C). Relative odds of exclusive breastfeeding and initiation of complementary feeding at 6 month in the comparison district in rural Uttar Pradesh, India (2004–06) ANC: Antenatal checkups received; AWW: Anganwadi worker; p-value for significant at: *p<0.05. **p<0.01 and ***p<0.001 (Table D).
(DOCX)

## Abbreviations

ANC: Antenatal checkups received
ANM: Auxiliary Nurse Midwives
AWC: Anganwadi center
AWW: Anganwadi Worker
BCC: Behavior change communication
BF: Breastfeeding
CARE: Cooperative for Assistance and Relief Everywhere
CBO: Community-based Organization
CF: Complementary-feeding
Comp: Comparison
GOI: Government of India
ICDS: Integrated Child Development Services
IFA: Iron-folic acid
INHP II: Integrated Nutrition and Health Program
Int: Intervention
JHBSPH: The Johns Hopkins Bloomberg School of Public Health (JHBSPH)
KGMU: King George Medical University
LMP: Last Menstrual Period
NGO: Non-government Organization
PHC: Primary Health care center
RCH: Reproductive and Child Health
SF: Supplementary Food
SN: Supplementary Nutrition
