# Supplementary material for: Non-governmental organization facilitation of a community-based nutrition and health program: Effect on program exposure and associated infant feeding practices in rural India
Source: PLoS One. 2017 Sep 14;12(9):e0183316. doi: 10.1371/journal.pone.0183316 (PMC5598933; doi:10.1371/journal.pone.0183316)
Supplement: S1 File — (DOC) [file pone.0183316.s001.doc]

# S1

# CARE India Nutrition Evaluation Research Project

Cohort Research Protocol

January 10, 2007

**Title:** Evaluation Research of the Nutrition Interventions in the INHP II areas of CARE India

**Collaborating Institutions:**

CARE India

Johns Hopkins University (JHU), Bloomberg School of Public Health

King Georges Medical University (KGMU)

### Investigators:

### CARE: Usha Kiran, Deepika Nayar Chaudhery, DS Panwar

### JHU: Veena Singh, Michele L. Dreyfuss, Laura E. Caulfield, Abdullah H. Baqui, Gary Darmstadt, Saifuddin Ahmed, Robert E. Black, Mathuram Santosham

KGMU: Vinod K. Srivastava, Ramesh C. Ahuja

## Background

India is the world’s largest contributor to child malnutrition. Approximately 50% of all children are underweight and about three-fourths are anemic. Maternal undernutrition and anemia are also very common. About 36% of Indian women are classified as underweight and likely to suffer from chronic energy insufficiency according to body mass index (BMI). The prevalence of maternal anemia is about 52%.

Despite decades of continued programmatic efforts, levels of malnutrition in India remain high. The proposed ER, therefore, seeks to:

1. Implement and evaluate the impact of a package of services designed to improve the nutritional status of mothers and children, and
2. Build on the lessons learned through these programmatic efforts.

*INHP-II ‘Basic Package’*

CARE-India's Integrated Nutrition and Health Program supports the efforts of the GOI to improve nutrition status of women and children in India through the Integrated Child Development Services program. The INHP-II “basic” package of interventions is, thus, focused on reducing the prevalence of childhood underweight and anemia within the framework of existing national programs and policies. This basic package is ultimately scheduled for large-scale implementation in 8 additional states as part of INHP-II.

The package (see Table 1 for details) includes prenatal nutrition, early initiation of and sustained exclusive breastfeeding for six months, continuation of breastfeeding with appropriate complementary feeding from 6-24 months (including feeding during and after illness), and iron and vitamin A supplements for children. This is part of a broader package of essential child health and nutrition interventions that also include newborn health and routine immunization.

A specific thrust of INHP-II is to address system constraints to program effectiveness within the public health system. These include inadequate staffing and supervision; interruptions in supplies; lack of coordination amongst programmatic partners; and poor information systems for management. INHP-II’s premise is that strengthening the capacity of service providers and communities will enhance the effectiveness of programs. Enhanced efficiency of service providers is, in turn, expected to promote improved dietary practices during pregnancy, lactation and infancy, timely immunization, optimal breastfeeding and young child feeding practices, and appropriate micronutrient supplementation. The service providers referred to here include Anganwadi Workers (AWWs) and Auxiliary Nurse Midwives (or ANMs). Community capacity is to be strengthened mainly by building capacity of ‘change agents’ and Community Based Organizations (CBOs). It is expected that these key capacity-building interventions will improve maternal and child nutritional status.

This proposal describes the plan for evaluating the impact of this basic package. This will be compared to a control arm in which the Government of India’s (GoI’s) Integrated Child Development Services (ICDS) program is operational.

Although integrated programs are not uncommon, few rigorous evaluations of such programs have been conducted. The ER will therefore make a valuable contribution to current knowledge on program effectiveness.

*Is program customization necessary for different regions in India?*

The economic and cultural heterogeneity of the Indian population raises concerns about whether programmatic efforts should be tailored to different regional settings in order to reduce maternal and child malnutrition. These concerns about program design are bolstered by the fact that previous programmatic efforts in the poorer states in India have not demonstrated improvements in child nutritional status. This is despite evaluations demonstrating the strength of the program implementation and evidence of behavior change. This raises two related questions:

1. Given the scope and density of such programs, are they likely to be more effective in areas where constraints to adequate child growth are less formidable?
2. Alternatively, is this a problem related to evaluation time-frame constraints? In other words, is it that program effectiveness is more easily demonstrated within a limited time frame in areas where constraints to adequate child growth are less formidable?

The proposed ER, therefore, seeks to implement and assess INHP-II activities in 2 Indian states that provide distinct ecological settings─ Uttar Pradesh (UP) and Andhra Pradesh (AP). UP has high rates of child undernutrition and infant mortality. In addition, UP is the location of the Newborn Care Evaluation Research Project that will bring additional supervisory resources and therefore facilitate close supervision of the sites. AP, on the other hand, has better nutritional status compared to all India figures, a receptive state government, high capacity to operationalize and monitor implementation of the ER activities and high potential uptake of ER results.

Table 1. Key components of the Basic Nutrition Package of INHP

| **Life Cycle Period** | **Basic Package** |
| --- | --- |
| Pregnancy | *3 contacts*   - Iron Folic Acid (IFA large) supplementation - Rest for 1-2 hours - Dietary advice - Consume one additional meal everyday - Consume all available foods in the house - Consume Supplementary Nutrition (SN) |
| Birth- 28 days | *3+ contacts at 0, 3 and 7 days*   - Initiate breastfeeding within 1 hour - Exclusive breastfeeding - Make sure baby is getting as much milk as possible, e.g. correct position and attachment - Feed from one breast until soft and then switch to the other in order for child to receive fore and hind milk   For newborn who does not feed vigorously; provide expressed breast milk with clean cup and spoon as long as breastfeeding is not adequately vigorous; frequent feeding   - Dietary advice for mother - Consume one additional meal everyday - Consume all available foods in the house   Consume SN |
| 1-5 months | *3 contacts at 1.5, 2.5 and 3.5 months*   - Exclusive breastfeeding for 6 months - Dietary advice for mother - Consume one additional meal everyday - Consume all available foods in the house - Consume SN |
| 6-8 months | *1+ contact*   - Continued breastfeeding - Appropriate Complementary Feeding - Initiate complementary foods: rice, dal, *suji*, banana etc. - Start with few teaspoons and gradually increase the quantity - Child should be fed 1/2 *katori* of soft mashed food 3 times a day, with 1/2 to 1 tsp of oil added - Feed the child yourself from a separate plate or bowl to assess how much the child has eaten; interact with the child - During illness breastfeed more frequently, provide fluids - After illness give more food at each sitting and feed more frequently until the child has regained weight |
| 9-11 months | *1+ contact*   - Continued breastfeeding - Appropriate Complementary Feeding - Child should be fed 1/ 2 *katori* of soft mashed food (rice, *dal*, vegetables, green leafy vegetables, fruits. Milk products, egg , meat , fish if available and feasible) 4 times with 1 tsp of oil added - Feed the child yourself from a separate plate or bowl to assess how much the child has eaten; interact with the child - During illness breastfeed more frequently, provide fluids - After illness give more food at each sitting and feed more frequently until the child has regained weight   Vitamin A and measles immunization at 9 months |
| 12-24 months | *2+ contacts*   - Continued breastfeeding - Appropriate Complementary Feeding - Child should be fed 3/4 *katori* of soft mashed food (rice, *dal*, green leafy vegetables, other vegetables, fruits. Milk products, egg , meat , fish if available and feasible) 4-5 times a day with 1-2 tsp oil - Feed the child yourself from a separate plate or bowl to assess how much the child has eaten; interact with the child - During illness breastfeed more frequently, provide fluids - After illness give more food at each sitting and feed more frequently until the child has regained weight - Vitamin A at 18 months - Give iron supplements beginning 12 months {GOI policy, available for about 50% of children} |

(Note: The key difference between the control (ICDS) and intervention arms is one of execution and coordination)

## Research Objectives

The main objective of the evaluation is to determine the effectiveness of the “basic” package of INHP-II interventions in terms of reducing child undernutrition of the child in the first 24 months of life. The effectiveness of this ‘basic’ package is to be compared to that of the ICDS package.

A second objective is to make a preliminary assessment of whether the INHP-II strategy is more suited by design to reducing 0 to 24 months child undernutrition only in ecological settings with relatively low economic and cultural constraints to reducing malnutrition. To arrive at an understanding of this, the proposed ER will examine differential impact of INHP-II in a relatively low-constraint ecological setting in India, AP, and a relatively high-constraint setting, UP.

## Proposed Evaluation Research Design

To address the objectives of the evaluation, three evaluation components are proposed (see Figure 1).

1. A pre-test, post-test of control versus program areas (or quasi-experimental design) will be used to assess the effectiveness of the intervention package in improving the nutritional status of children 0-24 months of age. The outcomes of interest are child anemia and underweight. As shown in Figure 1, cross-sectional surveys will be conducted among mothers and children 0-24 months of age in each arm in each site.
2. A collection of methodologies will be implemented to evaluate the process of implementation of the intervention in each site in terms of coverage, dose and fidelity.
3. A longitudinal design will be employed in UP to follow a cohort of women from late pregnancy and their children through (at least) 18 months of age to further examine program effectiveness as well as the specific programmatic experiences as they evolve over time.

***Relative Roles/ Collaborative Partners***

The INHP-II package will be implemented by CARE India. The evaluation methodology has been designed by Johns Hopkins University (JHU). The evaluation will be implemented by Johns Hopkins University (JHU) in partnership with CARE India and King Georges Medical University (KGMU) in Lucknow, India. The evaluation is expected to be conducted through contracts with survey research firms in UP and AP. JHU will conduct and support the analysis of data in both AP and UP.

**Figure 1. CARE India Evaluation Framework for INHP**

**Baseline Survey***

Cross-sectional

2 arms X 2 sites

12/03 – 2/04

3200 respondents per arm

**

**Final Survey***

Cross-sectional

2 arms X 2 sites

2-3/06

3200 respondents per arm

**Program Implementation**

**2/2004-1/2006 (24 months)**

**Process Evaluation**

Semi-annual adequacy surveys and report abstraction

***Survey measures**

Maternal/child anthropometry

Maternal/child morbidity

Maternal dietary pattern

Maternal hemoglobin status

Child feeding pattern

SES

Health care utilization

Adherence

Exposure to program elements

****Cohort measures**

Maternal/child anthropometry

Maternal/child morbidity

Child feeding pattern

Child dietary intakes

SES

Health care utilization

Adherence

Exposure to program elements

Contacts, advices

Immunization

## Sampling Frame

The sampling strategy for the evaluation is as follows. In both AP and UP, an INHP district will be selected as an intervention area while a similar non-INHP (ICDS) district will serve as comparison, giving a total of two districts sampled per state. In UP, Barabanki has been selected as the intervention district and Unnao as the control district. In AP, Karimnagar has been selected as the intervention district and Rangareddy as the control district.

The implementation approach of INHP is that of demonstrating the effect of a technical interventions package delivered through a set of best practices that are designed to maximize effectiveness in improving nutrition and health of children, and the subsequent systematic replication of these best practices.

Approximately 10% of the *Anganwadi* centers[[1]](#footnote-2) (AWCs) “covered” by INHP are demonstration sites. A demonstration site is characterised as an AWC which holds a Nutrition and Health Day, has a Community Based Organization (CBO) which is engaged in nutrition and health activities, has active change agents, has other need-based innovations which serve as the learning ground for the project. Replication is undertaken through focused and deliberate capacity building of government counterparts and systems strengthening in the remaining 90 % areas.

INHP has so far demonstrated four best practices, which have been found to be effective for service delivery and bringing about behaviour change. These are:

- Block Level resource mapping (BLRM)[[2]](#footnote-3)
- Nutrition and Health Days (NHDs)[[3]](#footnote-4)
- Change agents (CAs)[[4]](#footnote-5)
- Community Based Monitoring System (CBMS)[[5]](#footnote-6)

In this context INHP has laid out the year-wise milestones for replication. Therefore, while conducting the evaluation research, it is crucial to identify areas (blocks, sectors and AWCs) that will receive the project interventions through the replication mode. These areas identified for possible replication should then serve as a universe for sample selection, contrary to the entire district serving as a universe for sample selection.

In an intervention district, following steps will be followed to establish the universe for sample selection:

- As a first step, blocks will be identified where the respective ICDS block officials {Child Development Project Officer (CDPO)} are willing to undertake replication. It is likely that out of 10-14 blocks in the district, about 4-5 blocks are selected.
- Subsequently the respective CDPOs will identify next level of functionaries named as sector supervisors that can undertake replication in their areas. A block generally comprises of 3 to 5 sectors and therefore 2 to 3 sector supervisor's areas will be further identified for possible replication.
- As each sector comprises of 20 to 25 AWCs, the respective sector supervisors will then identify *Angawadi* Workers (AWWs) that can undertake replication activities within their AWCs area. It is expected that each identified sector supervisor will have at least 10 to 15 AWWs and their respective AWCs identified as replication sites.
- Therefore, in a district, it is likely that about 150 to 250 AWCs will be identified for replication for the first phase of replication. These selected AWCs can then serve as a universe for sample selection for establishing the baseline for the nutrition evaluation research.
- The AWCs selected through this procedure will be those that receive project intervention for the maximum duration of time as they will be the first to be identified as replication sites.

On the other hand, in the control district, any four blocks can be randomly selected and one sector per block will be randomly selected to get the desired number of AWCs.

***Sampling Design***

**Component 1: Cross-sectional evaluation surveys**

As shown in figure 1, a survey will be conducted during January 2004 - February 2004 for the purposes of providing baseline information on the comparability of the study arms in each of the two study sites. A second survey is planned during January-March 2006 to assess and compare key program indicators and nutritional outcomes across sites. The post-test survey will be conducted at the beginning of 2006, allowing about 24 months for the intervention period before the evaluation, and 12 months afterward for data analysis and dissemination of results. No mid-term evaluation is planned due to cost considerations; rather we intend to rely on information gathered from the cohort study and the process evaluation. A survey research firm will be identified to conduct these surveys in each site, as is being done in the Neonatal Care ER.

**Sample size for baseline and endline surveys**

Sample size is calculated to detect differences in various nutrition outcomes between each intervention district and its comparison district. Assumptions in making these calculations included a statistical power of 80% to detect a difference and a significance level of 95% (alpha = 0.05). The sample size is not increased to account for a design effect, which in nutrition studies has been small or trivial. The sample size estimations for children is based on three primary outcomes: shift in average z-scores in length and weight, stunting and anemia. Calculations for the primary outcomes for children and pregnant women are found in below.

**Table 2.1. Target sample size calculations for primary nutritional outcomes in cross-sectional surveys at baseline and 24 months**

| Outcome Measure | SD or P^0^ | Detectable difference in means by intervention arm | Sample size  per intervention arm* |
| --- | --- | --- | --- |
| Nutritional status  Children  Shift in average z-score  12-17 months  18-23 months  Stunting (%)  12-23 months | 1.1  1.1  60% | .18 z-score  .18 z-score  Absolute reduction 6% | 587 ~600  587 ~600  1101 ~1200 |
| Anemia  Children 12-23 months    Pregnant women | 80%  50% | Absolute reduction 6%  Absolute reduction 8% | 772 ~800  633 ~800 |

*Estimated sample size rounded to nearest ‘00.

We need approximately 1200 children aged 12-23 months for nutritional status outcomes and at least 800 children for anemia. The NFHS-2 data show that the prevalence of stunting (height-for-age <-2 SD of median of reference population) among children 12-23 months was 57.5%. We estimated a minimum sample size requirement of 1101 children for the detection of 6% absolute reduction in stunting from a 60% prevalence of stunting at baseline. We rounded the target sample to 1200 children aged 12-23 months. The target 1200 sample size is also sufficient to detect a change of at least 0.18 z-score units for anthropometric z-scores.

NFHS-2 reports that the prevalence of anemia among children aged 12-23 months nationally was 77.7% (children aged <3 years: 74.3% nationally, 73.9% in Uttar Pradesh, and 72.3% in Andhra Pradesh). We estimate that a sample of 772 children could detect a 6% reduction in anemia from the assumed 80% prevalence at baseline among children aged 12-23 months. However, recruitment for hemoglobin assessment includes all children 12-23 months of age who will be enrolled in the surveys for anthropometry and an interview, yielding a larger sample. For women of reproductive age, the prevalence of anemia was 51.8% nationally (48.7% in Uttar Pradesh and 49.8% in Andhra Pradesh). We estimate a minimum sample size requirement of 633 (~800) pregnant women to detect a 8% reduction in anemia prevalence.

Sample size for adequacy surveys

For the 2 adequacy surveys, we target 100 currently pregnant women and 500 mothers of children 0-23 months of age for interviews. The purpose of the adequacy surveys is to monitor the progress and implementation of the INHP-II intervention for various process indicators (e.g., home visits received, antenatal iron-folic acid supplements received), and statistical power is not a primary consideration in sample size estimation. However, we estimate the required sample size of at least 97 currently pregnant women to estimate the process indicators with 10% margin-of-error, and round the sample size to 100 pregnant women for the adequacy surveys. With a target sample of 500 mothers of children 0-23 months, we expect to detect at least a 9% difference in program coverage between the arms.

Based on population size, we need 24 and 13 AWC areas in AP and UP districts, respectively, to meet the sample size requirements.

**Sample Selection**

In each of the 4 districts in the evaluation, rural blocks were selected for the evaluation surveys. In UP state, the intervention district of Barabanki has 16 blocks, and CARE worked in 14 of the 16 blocks in the district, all of which were rural. The evaluation study is planned for the 10 blocks where CARE has been able to start their replication strategy immediately as the other 4 blocks has too many sector supervisor vacancies. The comparison district of Unnao has 16 blocks also, 15 of which are rural. Nine blocks will be randomly selected to represent this district in the evaluation. In AP state, each district selected for the evaluation has 15 blocks. In the intervention district of Karimnagar, CARE works in 9 of the 15 blocks, and the evaluation will be conducted in these 9 CARE blocks. In the comparison district of Rangareddy, 8 blocks will be selected randomly for the purpose of this evaluation.

Each block contains 3-6 sectors, an area with 15-25 AWC and an estimated population of 20,000-25,000 persons. In the intervention districts in each state, the sampling universe for survey sample selection include only those sectors and AWC areas that were participating in CARE’s INHP-II program as replication sites. All sectors in the selected blocks will have AWC areas participating in the intervention. In the comparison districts, the sampling universe included all functioning AWC from all sectors of the selected blocks in the district.

Baseline survey

We will use multi-stage survey sampling design to achieve the targeted sample of children and women.

*Uttar Pradesh.* In the intervention district (Barabanki), 2 sectors will be randomly sampled from each block (n=20 sectors). In the comparison district (Unnao), 2-4 sectors will be randomly sampled per block in proportion to the number of AWC per block (n=20 sectors). In both districts, 3 AWC areas will be randomly sampled from each selected sector (n=60 AWC). All the women identified will be interviewed from the sampled AWC areas.

*Andhra Pradesh.* In both districts, 2-3 sectors per block will be sampled in proportion to the number of AWC per block (n=20 sectors), and 4-6 AWC areas will be randomly sampled per sector (n=110 AWC). All the women identified will be interviewed from the sampled AWC areas.

Endline survey

The same sample selection process used at baseline will be employed again at endline.

Adequacy surveys

In all districts, one sector will be randomly sampled from each block for selection of the Adequacy Survey sample. In AP districts, 2-3 AWC per sector will be sampled in proportion to the number of AWC per block for a total of 24 selected AWC areas. In UP districts, 1-2 AWC per sector will be sampled in the same manner for a total of 13 selected AWC areas. The same sample of AWC areas will be used for both adequacy survey rounds to allow for tracking of program exposures in the same AWCs over time.

**Study Instruments**

The study will include three separate instruments:

1. Sociodemographic Questionnaire: This questionnaire will be administered to pregnant women and mothers of children 0-23 months. It will collect data on the respondents sociodemographic characteristics including pregnancy history, age, education, religion, caste, parity, birth intervals, occupation, work history, land ownership, source of income, and basic household amenities.
2. Currently Pregnant Women’s (CPW) questionnaire: This questionnaire will be administered only to women who were pregnant at the time of survey. It will collect information on a wide variety of issues including antenatal care utilization, health behaviors during pregnancy, micronutrient supplementation, dietary intake and practices, rest and workload, and nutritional status.
3. Mothers’ (MOM) questionnaire: This questionnaire will be administered to women with children 0-23 months of age. It will collect information on similar topics as those covered in the pregnant women’s questionnaire. In addition, it will include questions on delivery, postpartum contacts with service providers, infant and child feeding practices and dietary intake, immunization, micronutrient supplementation, and maternal and child nutritional status.

**Data Collection**

The evaluation involve 4 rounds of cross-sectional survey data collection: the baseline survey in January-February 2004, the Adequacy I survey in January 2005, the Adequacy II survey in July-August 2005, and the endline survey in January-March 2006. At each survey round, we will conduct interviews with currently pregnant women and mothers of children 0-23 months of age. Anthropometric measures of weight and height/length will be obtained at the baseline and endline surveys. Hemoglobin measurement for assessment of anemia will be carried out among currently pregnant women and children 12-23 months of age at the baseline and endline surveys only. The surveys will be implemented by TNS with guidance from Johns Hopkins and CARE India investigators.

House listing

A hired survey research firm will conduct a household listing operation in all the sampled AWC areas selected for the samples at each survey round. The purpose of the household listing is to identify households with eligible women and children only. If more than one pregnant woman or mother of a child 0-23 months of age will be identified in a household, interviewers will select one eligible woman randomly from that household. The sample of AWC areas are selected with the expectation of identifying at least 2,400 mothers of children 0-23 months of age and 800 currently pregnant women in each district. For the adequacy surveys, the sample targets will be 500 mothers of children 0-23 months of age and 100 currently pregnant women. However, all eligible women from sampled AWC areas will be recruited for survey enrollment.

Training and field work

Training of interviewers and supervisors for each survey round will be carried out by TNS with on-site technical guidance from JHU, KGMU and CARE personnel. The interview teams will be of 4 interviewers, 1 supervisor and 1 field editor each. There will be six teams per state. The supervisors will be trained to take the weight and height/length measurements from women and children, and to collect a fingerprick blood sample for hemoglobin assessment. The interviewers and field editors will be trained to assist in both these tasks. The JHU field office in Lucknow will organize, train, and manage independent quality control teams to supervise the TNS interview teams and assure data quality. The surveys in Uttar Pradesh and Andhra Pradesh will be conducted simultaneously at all survey rounds.

# Component 2: Data Collection Methods for the Cohort Study

**Population**

A longitudinal design will be employed to assess the program effectiveness as well as specific programmatic exposure that evolves over time in Barabanki and Unnao districts of UP state only. Women in their third trimester of pregnancy will be identified and they will be followed from the time of delivery till the time their baby reaches 18 months of age or the study ends whichever comes first. In each intervention and comparison districts, three rural blocks will be purposively selected.

**Sample Size**

The targeted cohort sample size is 400 eligible women in each arm. Babies born to mothers in this cohort will be followed up until they are 18 months of age. Sample size calculations for the cohort study are presented in table 4.

Here the sample size is calculated to detect differences in dietary intakes between the intervention group and the control group. Assumptions in making these calculations included a power of 80% and a significance level of 95% (alpha = 0.05).

**Table 4.1 Sample size calculations for cohort study**

| Outcome Measure | SD or P0 | Detectable difference in means by intervention arm | Sample size  per intervention arm |
| --- | --- | --- | --- |
| Dietary intakes  Children  7 - 10 months  11 - 14 months | 200  200 | 75  85 | 150  150 |

**Table 4.2: Estimated sample size for different outcomes**

| Outcome variables | Proportion in unexposed* | Proportion in exposed | Power (1-β) | Type I error (α) | Sample Size |
| --- | --- | --- | --- | --- | --- |
| Initiation of BF | 0.24 | 0.40 | 0.80 | 0.05 | 304 |
| Exclusive BF | 0.46 | 0.60 | 0.80 | 0.05 | 398 |
| Initiation of CF | 0.08 | 0.18 | 0.80 | 0.05 | 354 |
| Weight for age  - 6 mo  - 12 mo  - 18 mo | 0.33  0.28  0.19 | 0.20  0.16  0.09 | 0.80  0.80  0.80 | 0.05  0.05  0.05 | 360  372  376 |
| Height for age  - 6 mo  -12 mo  - 18 mo | 0.19  0.33  0.62 | 0.09  0.20  0.48 | 0.80  0.80  0.80 | 0.05  0.05  0.05 | 376  360  396 |
| Vitamin A | 0.09 | 0.19 | 0.80 | 0.05 | 376 |

** Data source: NFHS-3, Uttar Pradesh, India, 2005-06 report*

**Sampling Strategy**

We got 12.2 pregnant women per AWCs in our baseline survey. Therefore, we estimate that there are approximately 5 (~40%) third trimester pregnant women in each AWC in UP. To enroll 400 3^rd^ trimester pregnant women in each district, we will recruit pregnant women from 81-85 AWCs in each district.

A multi-stage sampling strategy will be employed. In both the Intervention and control districts, 3 blocks each has been selected. The selected blocks are Nindura, Banki and Fatehpur blocks in Barabanki district and Nawabganj, Hasanganj and Asoha blocks in Unnao district. Selection criteria include accessibility from the district town, physical proximity (to the other selected blocks and the town), and the absence of other known large MCH nutrition and health projects (e.g. ICMR). In Barabanki, only blocks where CARE is actively implementing INHP-II replication sites were considered. In Unnao (control district), the comparability of a block's population size and number of AWCs was considered in addition to the other criteria mentioned above. There are 6-7 sectors per block in Barabanki and 5-6 sectors per block in Unnao district. Four sectors will be randomly selected from each of the 3 blocks in each district. Altogether, there are 135 and 192 AWCs in the selected blocks of the intervention and comparison districts respectively. Finally, we selected 6-7 AWCs per sector from both Barabanki and Unnao districts to get the sample size of 81-85 AWCs each for study inclusion.

In Barabanki, we selected 83 AWCs from the list provided by CARE. There were three AWCs where CARE intervention was not in place and hence were dropped. One AWC was further added as it’s name was changed but was a part of our previous CARE list and therefore once name was re-confirmed from the field; was included in the final AWCs list. We finally got a list of 81 AWCs to be included in cohort in Barabanki district.

From the list provided by the District Program Officer (DPO) in Unnao, we selected 84 AWCs. Three AWCs were actually hamlets of other selected AWCs. Therefore, we finally had a list of 81 AWCs in Unnao district also.

**Data Collection**

As stated earlier, the purpose of the cohort study is to provide information on the course/experience of a sub-sample of potential beneficiaries in UP from the time of entry into the program until time of exit when their children are 18 months of age (or the project ends).

In the cohort study the woman is followed from late pregnancy to delivery, and she and her child are followed every three months postpartum until 18 months of age. These are the critical periods when service providers are suppose to interact and provide specific advices and services to these mothers. These follow-up visits are timed to reflect changes in program emphasis regarding the mother’s and child’s nutrition. At month-12 and month-18 visits, additional 24-hour dietary recalls will be collected on a 10% sub-sample (approximately 96 mothers) to estimate within-subject variation in dietary intakes. This will enable the ER to detect smaller differences in intake than presented (refer Table 4 for estimates presented).

***Recruitment***

Eligible pregnant women will be recruited simultaneously at the time of house-listing. One of the team will take oral consent and conduct the interview and take height and weight measurements of the index mother. If there is more than one pregnant women in the household, both will be enrolled for the study. Teams will give one calendar to the respondent (eligible enrolled pregnant woman) with the central office phone numbers to make a call within 24 hours of delivery.

***Eligibility***

All 81 AWCs in both the study districts will be house-listed. Houses will be listed by writing numbers on the right hand of the each front entry/ door of households with a white chalk. Right-hand rule will be followed in listing. According to this rule, when teams will reach a village they well ascertain a central point which is generally the house of the head of the village or sometimes anganwadi centre (AWC). From there, teams will starts numbering (for eg. 99/ NER) the houses moving in the right hand direction. This strategy is employed to avoid missing any household in the village since these rural villages are not planned. Listed households will be enquired about pregnancy status of all the women living in the household. All the women identified in their third trimester of pregnancy will be invited to enroll in the study if they plan to remain in the same household in the immediate future.

***Eligibility criteria***

A comprehensive house-listing will be maintained. All the pregnant women identified for cohort enrollment will be queried to ascertain eligibility.

Women will be considered eligible if:

1. They are at least 24 weeks (completed 6 months/ 180 days) pregnant on the day of house-listing. Gestational age will be assessed by recall of the date of the last menstrual period (LMP) and by asking completed months of pregnancy. In case woman is unsure of her LMP; teams will be provided with local festival calendar that will help in recalling the tentative date of LMP.
2. They intend to remain in the program area (same village) during the duration of the study. Women who are visiting their parents’ home should be excluded unless it is determined that their permanent residence is also within the cohort sample area so they can be followed up at that alternate location. Women who report that they intend to visit their parents’ home for delivery or during the postpartum period can still be enrolled as long as their visit is not intended to be long-term (more than 3 months) and someone in their family is willing to inform our study team about the birth regardless of a woman’s location.
3. Pregnant daughters who are permanently (more than one year) staying with their parents will be considered eligible.

The study will be explained to the women, and if she agrees to participate will be considered eligible. Women will be recruited into the cohort subject to their consent to participate.

***Personnel/ Staffing***

There will be 8 field interviewers employed for the cohort component. They will work in teams of two, with two teams each covering Barabanki and Unnao Districts. These four teams will work in both the districts. Field teams will be coordinated and supervised by the Nutrition Project Coordinator with assistance from the Field Coordinator. Dr. VK Srivastava and his colleagues at KGMU will provide technical assistance to the Project Coordinator and her cohort team.

Each field team will require 2-3 days to identify and enroll eligible women in each AWC, so one team will be able to cover approximately 16-18 AWCs per month. Using 2 field teams, recruitment and enrollment is estimated to take 3 months. We expect approximately 5-6 births per AWC. Because follow-up data collection activities will begin during the enrollment period, a third team will be needed in each district during that time (3 months). Therefore, two temporary workers will be hired in each district during the enrollment period so 3 teams will be available. Each temporary worker will be paired with a permanent field worker to form the data collection teams. One pair of permanent team members will start the follow-up visits as soon as team gets the delivery information. Rest, two pairs of one temporary and one permanent team member will continue with house-listing and enrollment.

One data editor, data entry personnel, programmer/ data manager will be employed. They will be based in central location (i.e. Lucknow) and work with Project Coordinator for programming, data, editing, entry and cleaning. They will also provide logistic support during trainings.

***Follow-up***

All the pregnant women considered eligible at the time of recruitment/ enrollment and have consented to enroll in the study will be followed-up (FUP) postpartum. These recruited women and their infants will be continued to followed until study ends or the child is 18 months of age. If the index child expires follow-up will be terminated after the visit at which the child’s death is ascertained. FUP visits will be continued with the child and it’s caregiver even if the mother expires. If the participating woman leaves the catchment area, she will be coded as ‘moved away’ and her FUP visits will be discontinued. For a woman who is out of village temporarily, follow-up visits will be continued every three months until the women returns or it is confirmed that she has moved permanently. If any visit is ‘missed’ due to unavailability of mother; first two sections of the previous questionnaires will be filled during subsequent visit. At a given visit, no more than two questionnaires will be filled although day-7 questionnaire can be filled at any given visit. Women enrolled in the study can also discontinue their participation and drop out at any follow-up visit.

FUP visits will be scheduled to take place at the critical period immediately after programmatic exposure occurs. Therefore, first FUP visit is scheduled at seven days after child birth as programmatic interventions are scheduled in first seven days after delivery. After first visit at day-7, later visits will be scheduled every three months from the date of birth of index child (child being followed-up). A birth calendar will be generated on the basis of every child’s date of birth. This will help in scheduling FUP visit dates to avoid manual or calculation mistakes in scheduling these visits by the team members.

All the visits completed within 15 days of scheduled date of visit will be considered on-time. Every Saturday of the week, teams will come to the office and submit a form that will report the number of AWC surveyed, number of houses listed, number of pregnant women identified and number of FUP visits completed. A weekly plan for next week’s visits will also be submitted to coordinate and track the movement of the teams by project/ field coordinator.

***Tracking & supervision***

At enrollment, teams will fill a register for all the enrollments done in each AWC for both the districts separately. In these registers, pregnant women’s address, LMP, expected date of delivery and other contacts will be recorded by AWC name. These registers will be updated by each team everyday. This will provide a data base to cover all possible deliveries and avoid missing them since it will help teams to cover all the expected deliveries in a given AWC even if mother/ respondent doesn’t make a phone call to the Lucknow office. Each AWC will be re-visited at least once in every 15 day to identify the pregnancy.

**Data collected**

Questionnaires will be filled with the help of mother and only in cases where mother is not alive, caretaker of the child will help filling the questionnaires.

Figure above shows the categories in which data will be collected at different age ranges/ time interval for every child. Since women has been enrolled at various stages of pregnancy, the range is from 6 completed months to 9 months, there is chance of missing the information on interactions with service providers, advices given and health services utilization for the interviews completed at earlier stages of pregnancy. Therefore, we will be repeating the information on advice given and health services utilization during pregnancy while filling Day-7 questionnaires.

Anthropometry

- weight
- length

Breastfeeding/ infant feeding practices

Day-7

- initiation
- current breast-feeding

Month-6

- Breastfeeding
- Introduction of complementary foods

Health Advice

At each interview, we ask about health advice received using both unprompted and prompted questions.

***Data collected in Cohort:***

| TIME PERIOD | 6-9 mo | 6-9 mo | 7 day | 7 Day | 3 mo | 3 mo | 6 mo | 6 mo | 9 mo | 9 mo | 12-24 mo | 12 mo | 15  mo | 18 mo |
| --- | --- | --- | --- | --- | --- | --- | --- | --- | --- | --- | --- | --- | --- | --- |
| PROGRAM  COMPONENTS | Dietary advice,  SN, TT2,  100 IFA,  Rest and workload, Birth plan |  | Bfd. < 60 min.  Exclusive bfdg,  Colostrum  Feeding, prob.  Immun,  Maternal diet,  Rest & workload, SN |  | Exclusive bfdg,  Immun,  Maternal diet,  Rest,  SN |  | Continued on-demand bfdg,  Weaning, Quantity & quality,  Feeding during illness,  SN,  Maternal diet,  Rest,  SN |  | Continued on-demand bfdg,  Quantity & quality,  SN, Feeding during illness,  SN,  Vitamin A at 9 months,  Maternal diet,  Rest,  SN |  | Continued on-demand bfdg,  Quantity & quality,  SN, Feeding during illness,  SN,  Iron tablets  Vitamin A (18 mo)  Maternal diet,  Rest,  SN |  |  |  |
| EVALUATION |  |  |  |  |  |  |  |  |  |  |  |  |  |  |
| Vital Status of Mother & Infant |  |  |  | X |  | X |  | X |  | X |  | X | X | X |
| ♀Socio-demographic information |  | X |  |  |  |  |  |  |  |  |  |  |  |  |
| ♀ Health Advice Given During Pregnancy |  | X |  | X |  |  |  |  |  |  |  |  |  |  |
| ♀ Health Service Utilization During Pregnancy |  | X |  | X |  |  |  |  |  |  |  |  |  |  |
| ♀ Birth Preparedness |  | X |  |  |  |  |  |  |  |  |  |  |  |  |
| ♀ Maternal Diet |  | X |  |  |  | X |  | X |  | X |  |  |  |  |
| ♀ Rest and Workload |  | X |  |  |  | X |  | X |  | X |  |  |  |  |
| ♀ Anthropometry |  | X |  |  |  |  |  |  |  |  |  |  |  |  |
| Delivery |  |  |  | X |  |  |  |  |  |  |  |  |  |  |
| Health advice given |  |  |  | X |  | X |  | X |  | X |  | X |  | X |
| Breast-feeding Initiation |  |  |  | X |  | X |  |  |  |  |  |  |  |  |
| Infant Feeding Practices |  |  |  | X |  | X |  | X |  | X |  | X | X | X |
| Feeding and Care During Infant Morbidity |  |  |  | X |  | X |  | X |  | X |  | X | X | X |
| Repeat Dietary Recall in 24-hr Child Feeding |  |  |  |  |  |  |  |  |  |  |  | X |  | X |
| Immunization |  |  |  |  |  | X |  | X |  | X |  | X | X | X |
| Child Anthropometry |  |  |  | X |  | X |  | X |  | X |  | X | X | X |

1. **Training and standardization:**
   1. **Interviews**
2. **dietary recall**
3. **advice questions (prompted/ un-prompted, probing protocols, decision making for coding)**
   1. **Anthropometry**
4. **Validation sub-studies**
   1. **Hemo-cue**
   2. **24 hour dietary recall**

1. AWC is a focal point of service delivery of ICDS package of services at the village or community level, covering a population of 1000 population . The Anganwadi is managed by an AWW, who belongs to the same community [↑](#footnote-ref-2)
2. Block Level Resource Mapping (BLRM) is a key tool for participatory planning and monitoring of key nutrition and health issues. Key government staff and partners map the information at block level for analyzing trends and identifying the problems and develop solutions to improve the service delivery. Action plan with clear roles and responsibilities for both ICDS and Health departments is chalked out. [↑](#footnote-ref-3)
3. Nutrition and Health Day (NHD) is defined as a “set day, happening at least once a month, when take-home rations are distributed, an ANM visits the AWC and offers immunization and/or antenatal care services”. [↑](#footnote-ref-4)
4. Change Agents (CA) is an active, interested member of the community who acts as a promoter and monitor of health and nutrition practices in the neighborhood (one per 15-20 families) and who counsels families to promote positive health behaviors on a voluntary basis. [↑](#footnote-ref-5)
5. Community Based Monitoring System (CBMS) consists of a set of tools that are evolved through a participatory process that enables communities to monitor their own nutrition and health practices as well as the facilitates service providers to target and improve coverage of services. [↑](#footnote-ref-6)
