# Supplementary material for: Non-governmental organization facilitation of a community-based nutrition and health program: Effect on program exposure and associated infant feeding practices in rural India
Source: PLoS One. 2017 Sep 14;12(9):e0183316. doi: 10.1371/journal.pone.0183316 (PMC5598933; doi:10.1371/journal.pone.0183316)
Supplement: S2 File — Relative odds of optimal breastfeeding initiation practices in the intervention district in rural Uttar Pradesh, India (2004–06) p-value for significant at: *p<0.05. **p<0.01 and ***p<0.001 (Table A). Odds of initiation of breastfeeding within < = 1 hour of delivery, feeding colostrum and avoiding pre-lacteals in the comparison district in rural Uttar Pradesh, India (2004–06) ANC: Antenatal checkups received; PHC: Primary Health care center; AWW: Anganwadi worker; P-value for chi2 tests: < 0.05 (Table B). Relative odds of optimal infant feeding practices at 6 month of age in rural Uttar Pradesh, India (2004–06) ANC: Antenatal checkups received; AWW: Anganwadi worker; p-value for significant at: *p<0.05. **p<0.01 and ***p<0.001 (Table C). Relative odds of exclusive breastfeeding and initiation of complementary feeding at 6 month in the comparison district in rural Uttar Pradesh, India (2004–06) ANC: Antenatal checkups received; AWW: Anganwadi worker; p-value for significant at: *p<0.05. **p<0.01 and ***p<0.001 (Table D). (DOCX) [file pone.0183316.s002.docx]

**S2 File**

**Table A Relative odds of optimal breastfeeding initiation practices in the intervention district in rural Uttar Pradesh, India (2004-06)**

| **Characteristics** | **Breastfeeding practices** | | | | | |
| --- | --- | --- | --- | --- | --- | --- |
|  | **Initiation of bf <=1 hr** | | **Colostrum feeding** | | **Avoiding pre-lacteals** | |
|  | **Odds ratio** | **(95% CI)** | **Odds ratio** | **(95% CI)** | **Odds ratio** | **(95% CI)** |
| **Socio-demographic** |  |  |  |  |  |  |
| Wealth index quintiles^2^ |  |  |  |  |  |  |
| 1 | 1.0 |  | 1.0 |  | 1.0 |  |
| 2 | 1.21 | ( 0.51, 2.85) | 0.82 | (0.42, 1.58) | 1.25 | (0.57, 2.72) |
| 3 | 1.44 | ( 0.63, 3.28) | 0.80 | (0.42, 1.53) | 0.96 | (0.43, 2.11) |
| 4 | 1.04 | ( 0.44, 2.43) | 1.00 | (0.54, 1.87) | 1.19 | (0.56, 2.54) |
| 5 | 1.08 | ( 0.46, 2.54) | 0.70 | (0.37, 1.33) | 0.96 | (0.44, 2.10) |
| Maternal education | 1.0 |  | 1.0 |  | 1.0 |  |
| Literate | 1.56 | (0.93, 2.63) | 1.22 | (0.81, 1.85) | 1.48 | (091, 2.40) |
| Paternal education | 1.0 |  | 1.0 |  | 1.0* |  |
| Literate | 1.13 | (0.67, 1.91) | 1.29 | (0.86, 1.93) | 0.56 | (0.32, 0.97) |
| Religion |  |  |  |  |  |  |
| Muslim | 1.0 |  | 1.0 |  | 1.0 |  |
| Hindu | 1.24 | (0.67, 2.30) | 1.66* | (1.01, 2.71) | 2.38*** | (1.21, 4.68) |
| Caste |  |  |  |  |  |  |
| General/others | 1.0 |  | 1.0 |  | 1.0 |  |
| OBC | 1.14 | (0.47, 2.76) | 0.76 | (0.41, 1.40) | 0.73 | (0.35, 1.50) |
| SC/ST | 2.00 | (0.83, 4.80) | 1.06 | (0.57, 1.98) | 1.12 | (0.54, 2.34) |
| Place of delivery |  |  |  |  |  |  |
| Home/other place | 1.0 |  | 1.0 |  | 1.0 |  |
| Government/ private hospital | 1.12 | (0.59, 2.13) | 1.30 | (0.79, 2.12) | 1.95* | (1.12, 3.38) |
| Mother working outside home | 1.0 |  | 1.0 |  | 1.0 |  |
| Yes | 0.58 | (0.32, 1.05) | 0.93 | (0.61, 1.41) | 0.82 | (0.49, 1.38) |
| Infant’s gender |  |  |  |  |  |  |
| Female | 1.0 |  | 1.0 |  | 1.0 |  |
| Male | 1.60 | (0.96, 2.65) | 1.44 | (0.98, 2.14) | 1.32 | (0.83, 2.11) |
| **Physiological correlates** |  |  |  |  |  |  |
| Maternal age |  |  |  |  |  |  |
| <20yr | 1.0 |  | 1.0 |  | 1.0 |  |
| 20-34yr | 0.70 | (0.37, 1.34) | 0.74 | (0.44, 1.25) | 1.43 | (0.71, 2.86) |
| 35-49yr | 0.75 | (0.26, 2.13) | 0.47 | (0.19, 1.15) | 0.87 | (0.28, 2.71) |
| Gravidity^4^ | 1.0 |  | 1.0 |  | 1.0 |  |
|  | 0.85 | (0.45, 1.59) | 0.67 | (0.41, 1.09) | 0.56 | (0.32, 0.97) |
| Birth interval^5^ |  |  |  |  |  |  |
| None | 1.0 |  | 1.0 |  | 1.0 |  |
| <24m | 0.55 | (0.25, 1.23) | 0.83 | (0.48, 1.44) | 0.45 | (0.21, 0.95) |
| 24-47m | 1.13 | (0.65, 1.97) | 0.77 | (0.49, 1.21) | 0.79 | (0.47, 1.35) |
| 48+ | 0.62 | (0.20, 1.91) | 0.67 | (0.30, 1.48) | 0.69 | (0.27, 1.79) |
| Pre-term births^6^ | 1.0 |  | 1.0 |  | 1.0 |  |
|  | 0.75 | (0.36, 1.54) | 0.86 | (0.50, 1.47) | 1.19 | (0.65, 2.20) |
| **Health worker contacts** |  |  |  |  |  |  |
| Anganwadi worker |  |  |  |  |  |  |
| Home visit | 3.00*** | (1.79, 5.02) | 2.84*** | (1.89, 4.26) | 4.24*** | (2.59, 6.96) |
| Any contact | 3.04*** | (1.72, 5.38) | 2.99*** | (1.97, 4.53) | 4.58*** | (2.59, 8.10) |
| Auxilliary nurse midwife |  |  |  |  |  |  |
| Home visit | 2.39** | (1.42, 4.05) | 2.11*** | (1.37, 3.26) | 1.91* | (1.16, 3.14) |
| Any contact | 2.14** | (1.26, 3.66) | 1.98*** | (1.33, 2.96) | 1.82* | (1.12, 2.95) |
| **Health services utilization** |  |  |  |  |  |  |
| Antenatal check-up | 2.16** | (1.30, 3.59) | 1.55* | (1.05, 2.31) | 2.28*** | (1.42, 3.68) |
| Receipt of: |  |  |  |  |  |  |
| Supplementary nutrition | 3.32*** | (1.73, 6.39) | 1.69* | (1.11, 2.57) | 4.02*** | (2.15, 7.52) |
| Tetanus toxoid vaccination | 2.70** | (1.37, 5.31) | 2.47*** | (1.53, 3.97) | 2.80*** | (1.49, 5.25) |
| Iron-folic acid tablets | 2.861*** | (1.54, 5.31) | 2.72*** | (1.75, 4.24) | 3.14*** | (1.75, 5.61) |

p-value for significant at: *p<0.05. ^**^p<0.01 and ^***^p<0.001

**Table B Odds of initiation of breastfeeding within <= 1 hour of delivery, feeding colostrum and avoiding pre-lacteals in the comparison district in rural Uttar Pradesh, India (2004-06)**

| **Characteristics** | **Initiation of bf <=1 hr** | | **Colostrum feeding** | | **Avoiding pre-lacteals** | |
| --- | --- | --- | --- | --- | --- | --- |
|  | **Odds ratio** | **(95% CI)** | **Odds ratio** | **(95% CI)** | **Odds ratio** | **ratio (95% CI)** |
| **Socio-demographic characteristics** |  |  |  |  |  |  |
| Wealth index quintiles^2^ |  |  |  |  |  |  |
| 1 | 1.0 |  | 1.0 |  | 1.0 |  |
| 2 | - |  | 0.53 | (0.17, 1.71) | - |  |
| 3 | - |  | 0.47 | (0.14, 1.64) | 0.19 | (0.02, 1.71) |
| 4 | 1.10 | (0.15, 8.02) | 0.37 | (0.09, 1.46) | - |  |
| 5 | 4.00 | (0.78, 20.64) | 1.34 | (0.48, 3.71) | 0.68 | (0.15, 3.17) |
| Maternal education | 1.0 |  | 1.0** |  | 1.0*** |  |
| Literate | 2.43 | (0.69, 8.56) | 2.97 | (1.44, 6.12) | 16.52 | (2.01, 135.79) |
| Paternal education | 1.0 |  | 1.0 |  | 1.0 |  |
| Literate | 2.05 | (0.43, 9.80) | 1.15 | (0.53, 2.48) | 1.46 | (0.18, 12.05) |
| Religion |  |  |  |  |  |  |
| Muslim | - |  | 1.0 |  | - |  |
| Hindu | - |  | 1.79 | (0.23, 13.84) | - |  |
| Caste |  |  |  |  |  |  |
| General/others | 1.0*** |  | 1.0* |  | 1.0 |  |
| OBC | 0.12 | (0.02, 0.57) | 0.24 | (0.10, 0.63) | 0.31 | (0.07, 1.40) |
| SC/ST | 0.06 | (0.01, 0.46) | 0.39 | (0.17, 0.91) | 0.10 | (0.01, 0.87) |
| Place of delivery |  |  |  |  |  |  |
| Home/other place | 1.0** |  | 1.0 |  | 1.0*** |  |
| Government/private hospital | 6.83 | (1.83, 25.48) | 2.49 | (0.95, 6.54) | 33.39 | (6.46, 172.45) |
| Mother working outside home | 1.0 |  | 1.0 |  | 1.0 |  |
| Yes | 0.40 | (0.08, 1.90) | 0.83 | (0.40, 1.73) | 0.22 | (0.03, 1.80) |
| Infant’s gender |  |  |  |  |  |  |
| Female | 1.0 |  | 1.0 |  | 1.0 |  |
| Male | 0.52 | (0.13, 2.06) | 0.83 | (0.40, 1.73) | 0.43 | (0.09, 2.14) |
| **Physiological characteristics** |  |  |  |  |  |  |
| Maternal age |  |  |  |  |  |  |
| <20yr | 1.0 |  | 1.0 |  | 1.0 |  |
| 20-34yr | 0.61 | (0.15, 2.42) | 1.53 | (0.57, 4.09) | 0.79 | (0.16, 3.97) |
| 35-49yr | - |  | - |  | - |  |
| Gravidity^4^ | 1.0 |  | 1.0 |  | 1.0 |  |
|  | 0.82 | (0.17, 3.94) | 2.18 | (0.65, 7.35) | 1.46 | (0.18, 12.05) |
| Birth interval^5^ |  |  |  |  |  |  |
| None | 1.0 |  | 1.0 |  | 1.0 |  |
| <24m | 0.77 | (0.15, 4.05) | 0.60 | (0.21, 1.73) | 0.47 | (0.05, 4.23) |
| 24-47m | 0.72 | (0.17, 3.06) | 0.80 | (0.35, 1.85) | 0.60 | (0.11, 3.36) |
| 48+ | - |  | 0.71 | (0.19, 2.56) | 0.91 | (0.10, 8.34) |
| Pre-term births^6^ | 1.0 |  | 1.0 |  | 1.0 |  |
|  | 2.05 | (0.41, 10.16) | 0.22 | (0.03, 1.62) | 1.16 | (0.14, 9.84) |
| **Health worker contacts** |  |  |  |  |  |  |
| Anganwadi worker |  |  |  |  |  |  |
| Home visit | 1.09 | (0.23, 5.27) | 0.98 | (039, 2.48) | 2.73 | (0.64, 11.70) |
| Any contact | 0.37 | (0.05, 2.92) | 1.32 | (0.59, 2.97) | 2.10 | (0.49, 8.98) |
| Auxiliary nurse midwife |  |  |  |  |  |  |
| Home visit | 2.19 | (0.62, 7.70) | 1.26 | (0.60, 2.64) | 3.70 | (0.87, 15.73) |
| Any contact | 3.02 | (0.63, 14.44) | 0.78 | (0.38, 1.58) | 5.36 | (0.65, 43.95) |
| **Health services utilization** |  |  |  |  |  |  |
| Antenatal care check-up | 2.27 | (0.65, 8.01) | 1.26 | (0.60, 2.64) | 6.74* | (1.34, 33.87) |
| Receipt of: |  |  |  |  |  |  |
| Supplementary nutrition | 0.68 | (0.14, 3.25) | 1.94 | (0.93, 4.05) | 1.70 | (0.40, 7.23) |
| Tetanus toxoid vaccination | - |  | 0.88 | (0.42, 1.82) | - |  |
| Iron-folic acid tablets | 3.35 | (0.70, 15.98) | 0.77 | (0.38, 1.57) | - |  |

ANC: Antenatal checkups received

PHC: Primary Health care center

AWW: Anganwadi worker

P-value for chi2 tests: < 0.05

**Table C Relative odds of optimal infant feeding practices at 6 month of age in rural Uttar Pradesh, India (2004-06)**

| **Characteristics** | **Infant feeding practices** | | | |
| --- | --- | --- | --- | --- |
|  | **Exclusive breastfeeding**  **upto 6 mo** | | **Initiation of complementary feeding after 6 mo** | |
|  | **Odds ratio** | **(95% CI)** | **Odds ratio** | **(95% CI)** |
| **Socio-demographic variables** |  |  |  |  |
| Wealth index quintiles^2^ |  |  |  |  |
| 1 | 1.0*** |  | 1.0*** |  |
| 2 | 1.95 | (1.00, 3.83) | 1.25 | (0.64, 2.45) |
| 3 | 2.86 | (1.42, 5.77) | 1.45 | (0.75, 2.82) |
| 4 | 2.05 | (1.07, 3.94) | 1.47 | ( 0.77, 2.81) |
| 5 | 5.46 | (2.47, 12.04) | 3.53 | (1.81, 6.87) |
| Mother’s education | 1.0*** |  | 1.0*** |  |
| Literate | 2.29 | (1.37, 3.84) | 2.99 | (1.93, 4.62) |
| Father’s education | 1.0*** |  | 1.0* |  |
| Literate | 1.80 | (1.16, 2.78) | 1.60 | (1.06, 2.41) |
| Religion % (n) |  |  |  |  |
| Muslim | 1.0 | 1.0 | 1.0 |  |
| Hindu | 0.98 | (0.58, 1.63) | 1.10 | (0.68, 1.78) |
| Caste % (n) |  |  |  |  |
| General/others | 1.0*** |  | 1.0** |  |
| OBC | 0.38 | (0.15, 0.93) | 0.54 | (0.29, 1.03) |
| SC/ST | 0.28 | (0.11, 0.70) | 0.37 | (0.19, 0.71) |
| Mother working outside home | 1.0*** |  | 1.0** |  |
| Yes | 0.55 | (0.35, 0.86) | 0.51 | (0.33, 0.79) |
| Infant gender % (n) |  |  |  |  |
| Female | 1.0 |  | 1.0 |  |
| Male | 1.10 | (0.72, 1.70) | 1.14 | ( 0.77, 1.69) |
| **Physiological correlates** |  |  |  |  |
| Maternal age % (n) |  |  |  |  |
| <20yr | 1.0 |  | 1.0 |  |
| 20-34yr | 1.13 | (0.63, 2.02) | 1.27 | (0.73, 2.21) |
| 35-49yr | 1.89 | (0.68, 5.24) | 0.75 | (0.31, 1.79) |
| Gravidity^4^ | 1.0 |  | 1.0* |  |
|  | 0.79 | (0.45, 1.40) | 0.57 | (0.34, 96) |
| Birth interval %(n)^5^ |  |  |  |  |
| None | 1.0 |  | 1.0* |  |
| <24m | 0.80 | (0.44, 1.47) | 0.66 | (0.38, 1.14) |
| 24-47m | 0.81 | (0.49, 1.34) | 0.58 | (0.37, 0.93) |
| 48+ | 1.35 | (0.52, 3.49) | 0.31 | (0.13, 0.76) |
| Pre-term births % (n)^6^ | 1.0* |  | 1.0 |  |
|  | 0.51 | (0.30, 0.88) | 0.87 | (0.50, 1.49) |
| **Health worker contacts** |  |  |  |  |
| Anganwadi worker |  |  |  |  |
| Home visit | 0.54* | (0.32, 0.92) | 0.74 | (0.46, 1.18) |
| Any visit | 1.15 | (0.70, 1.89) | 0.84 | (0.54, 1.29) |
| Auxiliary nurse midwife |  |  |  |  |
| Home visit | 0.89*** | (0.56, 1.42) | 0.91 | (0.61, 1.35) |
| Any visit | 0.66 | (0.41, 1.07) | 0.73 | (0.49, 1.08) |
| **Health services utilization** |  |  |  |  |
| Antenatal check-up | 2.60*** | (1.61, 4.20) | 2.00*** | (1.34, 3.00) |
| Receipt of: |  |  |  |  |
| Supplementary nutrition | 0.89 | (0.57, 1.40) | 0.80 | ( 0.53, 1.21) |
| Tetanus toxoid vaccination | 1.66 | (1.05, 2.62) | 1.23 | ( 0.80, 1.90) |
| Iron-folic acid tablets | 1.22 | (0.79, 1.90) | 1.03 | ( 0.68, 1.55) |

ANC: Antenatal checkups received

AWW: Anganwadi worker

p-value for significant at: *p<0.05. ^**^p<0.01 and ^***^p<0.001

**Table D Relative odds of exclusive breastfeeding and initiation of complementary feeding at 6 month in the comparison district in rural Uttar Pradesh, India (2004-06)**

| **Characteristics** | **Exclusive breastfeeding**  **upto 6 mo** | | **Initiation of complementary**  **feeding after 6 mo** | |
| --- | --- | --- | --- | --- |
|  | **Odds ratio** | **(95% CI)** | **Odds ratio** | **(95% CI)** |
| **Socio-demographic variables** |  |  |  |  |
| Wealth index quintiles^2^ |  |  |  |  |
| 1 | 1.0 |  | 1.0** |  |
| 2 | 0.98 | (0.43, 2.22) | 0.84 | (0.40, 1.75) |
| 3 | 0.96 | (0.41, 2.22) | 1.01 | (0.47, 2.15) |
| 4 | 1.40 | (0.56, 3.51) | 2.61 | (1.21, 5.64) |
| 5 | 1.71 | (0.84, 4.58) | 2,64 | (1.24, 5.65) |
| Mother’s education | 1.0* |  | 1.0 |  |
| Literate | 2.05 | (1.05, 4.00) | 1.60 | (1.00, 2.56) |
| Father’s education | 1.0 |  |  |  |
| Literate | 1.21 | (0.69, 2.14) | 1.50 | (0.93, 2.42) |
| Religion % (n) |  |  |  |  |
| Muslim | 1.0 |  | 1.0 |  |
| Hindu | 0.54 | (0.12, 2.36) | 0.86 | (0.33, 2.23) |
| Caste % (n) |  |  |  |  |
| General/others | 1.0 |  | 1.0 |  |
| OBC | 0.56 | (0.24, 1.30) | 0.77 | (0.41, 1.44) |
| SC/ST | 0.81 | (0.34, 1.89) | 0.92 | (0.49, 1.71) |
| Mother working outside home | 1.0* |  | 1.0 |  |
| Yes | 0.56 | (0.32, 0.96) | 1.56 | (0.99, 2.45) |
| Infant gender % (n) |  |  |  |  |
| Female | 1.0 |  | 1.0 |  |
| Male | 1.02 | (0.59, 1.76) | 1.00 | (0.64, 1.55) |
| **Physiological correlates** |  |  |  |  |
| Maternal age % (n) |  |  |  |  |
| <20yr | 1.0 |  | 1.0 |  |
| 20-34yr | 1.28 | (0.66, 2.48) | 0.98 | (0.54, 1.77) |
| 35-49yr | 1.09 | (0.28, 4.30) | 0.75 | (0.23, 2.40) |
| Gravidity^4^ | 1.0 |  | 1.0 |  |
|  | 0.71 | (0.32, 1.58) | 0.57 | (0.31, 1.06) |
| Birth interval %(n)^5^ |  |  |  |  |
| None | 1.0 |  | 1.0 |  |
| <24m | 0.87 | (0.42, 1.79) | 0.65 | (0.36, 1.18) |
| 24-47m | 1.00 | (0.52, 1.93) | 0.60 | (0.35, 1.02) |
| 48+ | 1.02 | (0.39, 2.71) | 0.58 | (0.27, 1.22) |
| Pre-term births % (n)^6^ | 1.0 |  | 1.0 |  |
|  | 0.65 | (0.31, 1.39) | 0.68 | (0.34, 1.38) |
| **Health worker contacts** |  |  |  |  |
| Anganwadi worker |  |  |  |  |
| Home visit | 1.59 | (0.68, 3.71) | 1.16 | (0.69, 1.96) |
| Any visit | 1.51 | (0.77, 2.94) | 1.15 | (0.73, 1.82) |
| Auxiliary nurse midwife |  |  |  |  |
| Home visit | 1.31 | (0.71, 2.42) | 1.38 | (0.88, 2.15) |
| Any visit | 1.88 | (0.87, 4.06) | 1.35 | (0.84, 2.15) |
| **Health services utilization** |  |  |  |  |
| Antenatal care check-up | 1.71 | (0.90, 3.23) | 1.38 | (0.86, 2.22) |
| Receipt of: |  |  |  |  |
| Supplementary nutrition | 0.90 | (0.49, 1.65) | 0.71 | (0.43, 1.19) |
| Tetanus toxoid vaccination | 1.91 | (1.11, 3.29) | 1.10 | (0.70, 1.74) |
| Iron-folic acid tablets | 1.06 | (0.62, 1.82) | 0.69 | (0.45, 1.08) |

ANC: Antenatal checkups received

AWW: Anganwadi worker

p-value for significant at: *p<0.05. ^**^p<0.01 and ^***^p<0.001
